# Analyzing and Validating the Prognostic Value of a TNF-Related Signature in Kidney Renal Clear Cell Carcinoma

**DOI:** 10.3389/fmolb.2021.689037

**Published:** 2021-05-28

**Authors:** Wenhao Zhang, Changjiu Li, Fanding Wu, Ning Li, Yuwei Wang, Yixuan Hu, Tiantian Fang, Hui Yuan, Huadong He

**Affiliations:** ^1^ The Fourth School of Clinical Medicine, Zhejiang Chinese Medical University, Hangzhou, China; ^2^ Department of Urology, Affiliated Hangzhou First People’s Hospital, Zhejiang University School of Medicine, Hangzhou, China; ^3^ Department of Urology, Affiliated Hangzhou First People’s Hospital, Nanjing Medical University, Hangzhou, China; ^4^ School of Computer and Information Engineering, Zhejiang Gongshang University, Hangzhou, China

**Keywords:** signature, kidney renal clear cell carcinoma, biomarker, prognosis, immune

## Abstract

**Background:** Kidney renal clear cell carcinoma (KIRC) has the highest incidence rate in renal cell carcinoma (RCC). Although bioinformatics is widely used in cancer, few reliable biomarkers of KIRC have been found. Therefore, continued efforts are required to elucidate the potential mechanism of the biogenesis and progression of KIRC.

**Methods:** We evaluated the expression of tumor necrosis factor (TNF) family genes in KIRC, and constructed a prognostic signature. We validated the signature by another database and explored the relationship between the signature and progression of KIRC. We assessed the prognostic value, immune infiltration, and tumor mutation burden (TMB) of the signature in KIRC.

**Results:** We selected four key genes (*TNFSF14*, *TNFRSF19*, *TNFRSF21*, and *EDA*) to construct the TNF-related signature. We divided the KIRC patients into high- and low-risk groups based on the signature. Patients with higher risk scores had shorter overall survival and worse prognosis. With another database, we validated the value of the signature. The signature was considered as an independent risk factor. A higher level of risk score was relevant to higher level of immune infiltration, especially T regulatory cells, CD8^+^ T cells, and macrophages. The signature was also associated with TMB scores, and it may have an effect on assessing the efficacy of immunotherapy.

**Conclusion:** This is the first TNF-family-related signature of KIRC and we demonstrated its effectiveness. It played a significant role in predicting the prognosis of patients with KIRC. It also has the potential to become a powerful tool in guiding the immunotherapy of KIRC patients in clinical practice.

## Introduction

Kidney carcinoma ranks as the 16th most common cause of cancer mortality worldwide (Znaor et al., 2015). Renal cell carcinoma (RCC) is the most common type of renal carcinoma and is responsible for up to 90% of cases (Ljungberg et al., 2019). The main pathological type of RCC is kidney renal clear cell carcinoma (KIRC), which accounts for 70–80% of cases (Nerich et al., 2014). With the further exploration of the tumor microenvironment and the development of immunotherapy, the interaction between tumor and immune system has been studied in depth (Galon and Bruni, 2019). However, the cell phenotypes and corresponding molecular mechanism of KIRC have not been established (Du et al., 2017). There are few reliable biomarkers to predict prognosis and immunotherapeutic response. Therefore, continued efforts are required to elucidate the potential mechanism of the biogenesis and progression of KIRC.

The tumor necrosis factor (TNF) family comprises the 19 TNF ligands superfamily (TNFSF) and 29 TNF receptor superfamily (TNFRSF), and is one of the best-studied protein families over the past 3 decades (Feng, 2005). Previous research has shown that TNF is an inflammatory regulator that can activate immune cells through TNFR1 and TNFR2 (Al-Lamki et al., 2010). Besides, recent studies have found that a variety of cancers, including KIRC, are closely related to the TNF family (Croft et al., 2013). We assumed that KIRC has a potential relationship with the TNF family and performed this study for validation.

In this study, we evaluated mRNA expression data, clinical information, and mutation data of KIRC patients from the TCGA database. We constructed a prognostic multi-gene signature with differentially expressed genes of the TNF family and verified the efficacy of the signature. Functional enrichment analysis, immune infiltration, and tumor mutation burden (TMB) were used to explore the underlying mechanisms of the signature.

## Materials and Methods

### Acquisition of Patient Materials

The transcriptome profiles with HTSeq-FPKM format of KIRC patients were obtained from the TCGA database via the GDC portal (https://portal.gdc.cancer.gov/). The corresponding clinical data were also downloaded from the TCGA database. Data collected from the TCGA database were used as the training set. The transcriptome expression data and corresponding clinical information of KIRC patients obtained from the International Cancer Genome Consortium (ICGC) database (https://dcc.icgc.org/projects) were considered as the validation set. All patients with incomplete data were excluded.

### Construction of Signature and Survival Analysis

Cox’s proportional hazards regression model was utilized to construct the signature. We used univariate Cox analysis to identify the prognostic genes. Genes considered significant with a cutoff point of *p* < 0.05 were selected to built a stepwise Cox regression model. According to the result, we applied the following formula to calculated the risk score:

$$\left[\mathrm{Risk}\;\mathrm{score}=\overset{\text{n}}{\underset{\text{i}=1}{\sum }}\text{Coef}\left(\text{i}\right)*\text{x}\left(\text{i}\right)\right]$$

Coef (i) and x (i) represented estimated regression value. Patients were divided into high- and low-groups by the median risk score. A Kaplan–Meier curve was drawn using the R package “survival” to compare the survival difference of the two groups. Receiver operating characteristic (ROC) curve was drawn by the R package “survivalROC” to assess the predictive effect of the signature on overall survival (OS).

### Validation of the Signature

Validation data were downloaded from the ICGC database. The risk score of each patient was calculated with the same genes and coefficient score based on the signature. Kaplan–Meier and ROC curves were drawn to verify the predictive value of the signature.

### The Signature Acts as an Independent Risk Factor

Univariate analysis and stepwise Cox regression model were performed to explore whether the TNF-related signature could be an independent risk factor of other clinical characteristics (including age, gender, grade, and stage) in the TCGA database. Patients in the TCGA database were classified into age ≤65 years and >65 years subgroups, female and male subgroups, G1/2 and G3/4 subgroups, stage I/II and III/IV subgroups, and high- and low-risk subgroups. OS analysis via R package “survival” was utilized in every subgroup.

### Functional Enrichment Analysis

The KIRC patients were divided into high- and low-risk groups by the TNF-related signature. Then we applied gene ontology (GO) enrichment analysis to identify the biological processes. The Kyoto Encyclopedia of Genes and Genomes (KEGG) was utilized to establish the main signaling pathways regulated by the signature.

### Immune Cell Infiltration Analysis

The transcriptome gene expression data of KIRC patients downloaded from the TCGA database was normalized via “limma” package. Then CIBERSORT algorithm was utilized to evaluate the immune infiltration. The CIBERSORT was based on the known reference set which containing 22 leukocyte subtypes (LM22). Wilcoxon rank-sum test was performed to calculate the infiltration difference between high- and low- risk groups, and the result was exhibited by “vioplot” package.

### Tumor Mutation Burden Analysis

TMB was considered a measurement to calculate the total number of mutations in per million somatic genes. We downloaded the tumor mutation data of KIRC from the TCGA database and calculated the mutation rate of each sample via R package “maftools”. We further assessed the mutation discrepancy between high- and low-groups by Wilcoxon test.

### Statistical Analysis

All statistical analyses and generation of figures were performed by R software 4.0.2. *p* < 0.05 indicated significant effectiveness.

## Results

### Construction of Tumor Necrosis Factor-Related Signature by the Cancer Genome Atlas Database

**FIGURE 1:**
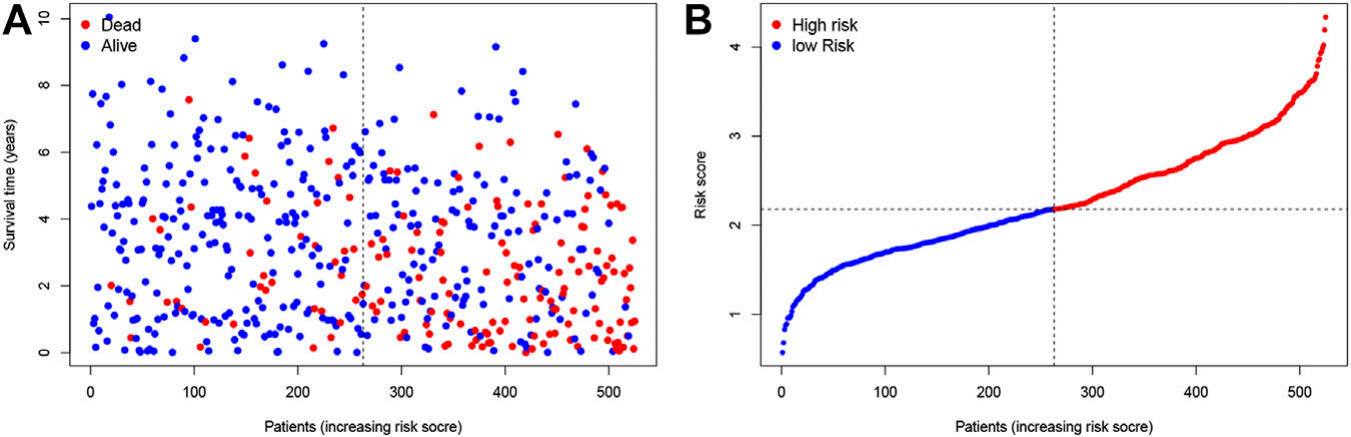
Construction of the TNF-related signature by the TCGA database. **(A, B)** The contribution of risk score and survival status.

**FIGURE 2:**
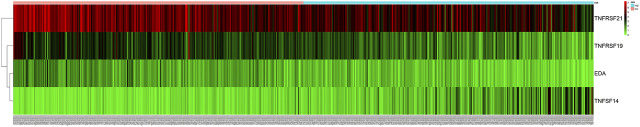
The relationship between survival status and gene expression of the signature. TNFSF14 was highly expressed in high-risk group. TNFRSF19, TNFRSF21, and EDA were highly expressed in low-risk group.

**FIGURE 3:**
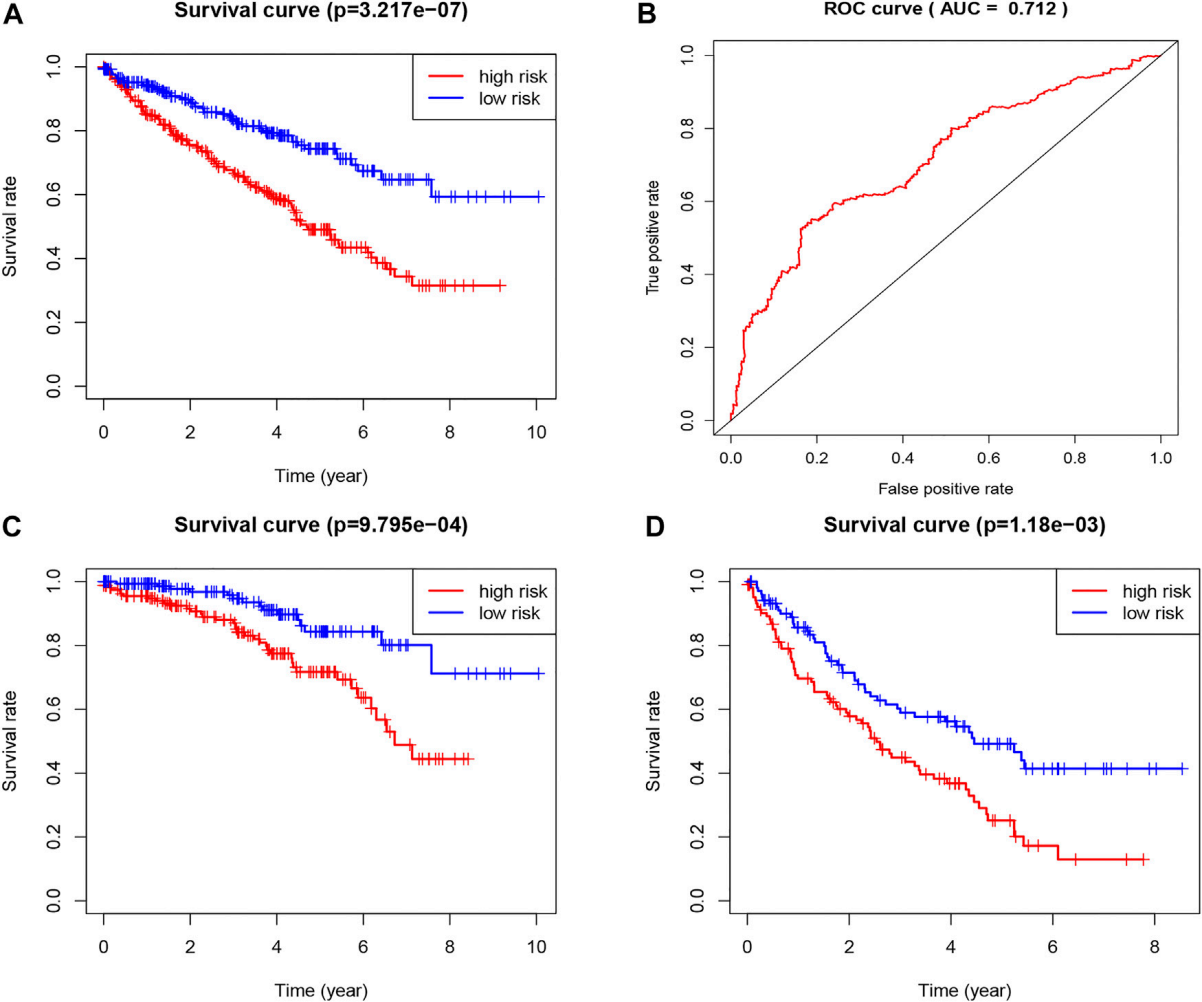
Construction of the TNF-related signature by the TCGA database. **(A)** Kaplan-Meier survival curve of OS in total KIRC patients that classified by the TNF-related signature into high- and low-risk groups. **(B)** ROC curve showing the values of the signature for OS among KIRC patients. **(C)** Kaplan-Meier survival curve of OS in early stage (I and II) KIRC patients that classified by the TNF-related signature into high- and low-risk groups. **(D)** Kaplan-Meier survival curve of OS in advanced stage (III and IV) KIRC patients that classified by the TNF-related signature into high- and low-risk groups.

We chose 47 TNF family genes. Table 1 showed the characteristics of all included patients. We performed a univariate Cox regression analysis and found 15 genes that contained seven TNF family genes and eight TNFRSF family genes (Table 2). We chose genes of *p* < 0.05 and built a stepwise Cox regression model to optimize the signature. Four genes were selected to construct the TNF-related signature: *TNFSF14* (TNFSF family), *TNFRSF19* (TNFRSF family), *TNFRSF21* (TNFRSF family), and *EDA* (TNFSF family). Among these four genes, *TNFSF14* was a high-risk factor (hazard ratio [HR] = 1.555, 95% confidence interval [CI] = 1.343–1.801), and *TNFRSF19* (HR = 0.678, 95% CI = 0.584–0.787), *TNFRSF21* (HR = 0.738, 95% CI = 0.638–0.853), and *EDA* (HR = 0.418, 95% CI = 0.303–0.577) were considered as low-risk factors. The risk score formula was formed by the expression level of the four genes and Cox coefficient: risk score = 0.25706 * TNFSF14 − 0.30544 * TNFRSF19– − 0.24573 * TNFRSF21 − 0.33039 * EDA. We divided the KIRC patients into high- and low-risk groups by the median risk score. The distribution characteristics of the four genes and the relevant risk score are shown in Figures 1, 2. Kaplan–Meier analysis was applied to assess the value in predicting OS of KIRC patients. Patients in the low-risk group had better OS (Figure 3A). The ROC curve of 5-years OS was plotted to show the prognostic accuracy of the TNF-related signature (AUC = 0.712) (Figure 3B). We divided the KIRC patients into early stage (I and II) and advanced stage (III and IV), and applied the TNF-related signature. Higher risk contributed to worse OS regardless of clinical stage (Figures 3C,D).

**TABLE 1:** Characteristics of patients with KIRC.

Characteristics	Variable	Total	Percentages (%)
Age	≦65	352	65.55
	>65	185	35.45
Gender	Male	346	64.43
	Female	191	35.57
Grade	Grade 1	14	2.61
	Grade 2	230	42.83
	Grade 3	207	38.55
	Grade 4	78	14.52
	G X	5	0.93
	Unknown	3	0.56
Stage	Stage I	269	50.09
	Stage II	57	10.61
	Stage III	125	23.28
	Stage IV	83	15.46
	Unknown	3	0.56
T	T1	275	51.21
	T2	69	12.85
	T3	182	33.89
	T4	11	2.05
N	N0	240	44.69
	N1	17	3.17
	NX	280	52.14
M	M0	426	79.33
	M1	79	14.71
	MX	30	5.59
	Unknown	2	0.37
Survival rate	Survival	367	68.34
	Dead	170	31.66

**TABLE 2:** 15 genes associated with patients’ OS.

Gene	HR	Z	*p* value
CD27	1.125	1.832	0.067
CD70	1.056	1.204	0.229
EDA	0.492	−4.750	<0.001
EDA2R	0.615	−3.935	<0.001
FASLG	1.229	1.922	0.055
TNFRSF9	1.223	2.222	0.026
TNFRSF11B	0.838	−2.400	0.016
TNFRSF18	1.744	5.008	<0.001
TNFRSF19	0.588	−5.906	<0.001
TNFRSF21	0.700	−4.448	<0.001
TNFSF4	1.150	1.515	0.130
TNFSF9	1.138	1.454	0.146
TNFSF13	0.576	−4.855	<0.001
TNFSF13B	1.419	4.132	<0.001
TNFSF14	1.588	6.145	<0.001

HR, hazard ratio; Z, Z tezt.

### Validation of the Tumor Necrosis Factor-Related Signature by International Cancer Genome Consortium Database

**FIGURE 4:**
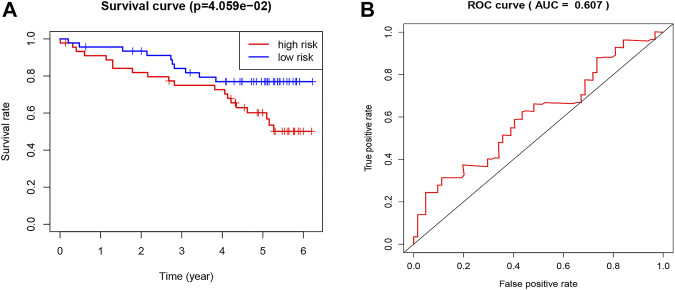
Validation of the TNF-related signature by the ICGC database. **(A)** Kaplan-Meier survival curve of OS in KIRC patients from ICGC database that classified by the TNF-related signature into high- and low-risk groups. **(B)** ROC showing the values of the signature for OS among KIRC patients in the ICGC database.

To validate the prognostic value of the TNF-related signature, we applied a new cohort downloaded from the ICGC database of KIRC patients. We used the same formula as the TCGA cohort to calculate the risk score of each patient and set the median score as the cutoff to divide the patients into high- and low-risk groups. The Kaplan–Meier curve, as expected, showed that the low-risk group had better OS (Figure 4A). The ROC curve of 5-year OS was plotted to validate the prognostic accuracy (AUC = 0.607) (Figure 4B).

### Independence of the Tumor Necrosis Factor-Related Signature as a Risk Factor

**FIGURE 5:**
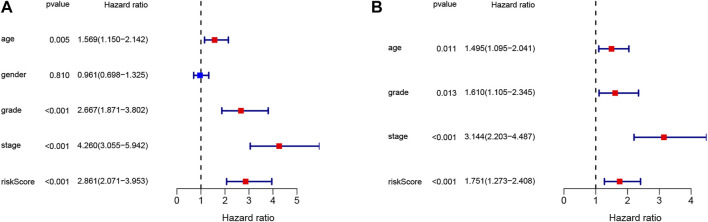
The signature identified as an independent risk factor. **(A)** The result of univariate Cox regression analysis. **(B)** The result of multivariate Cox regression analysis.

To confirm the independence of the TNF-related signature as a risk factor, we utilized univariate and multivariate Cox analysis in the TCGA database (Figures 5A,B). The pathological features explored included age, gender, stage, grade, and risk score. The high-risk group showed a difference in age (*p* = 0.011 HR = 1.495), grade (*p* = 0.013, HR = 1.610), stage (*p* < 0.001, HR = 3.144), and risk score (*p* < 0.001, HR = 1.751). The risk score was effective and the TNF-related signature was identified as an independent risk factor. The relationship between risk score and distribution of clinical characteristics were listed in the heatmap (Supplementary Figure S1).

### Investigation of the Biological Pathway About the Tumor Necrosis Factor-Related Signature

**FIGURE 6:**
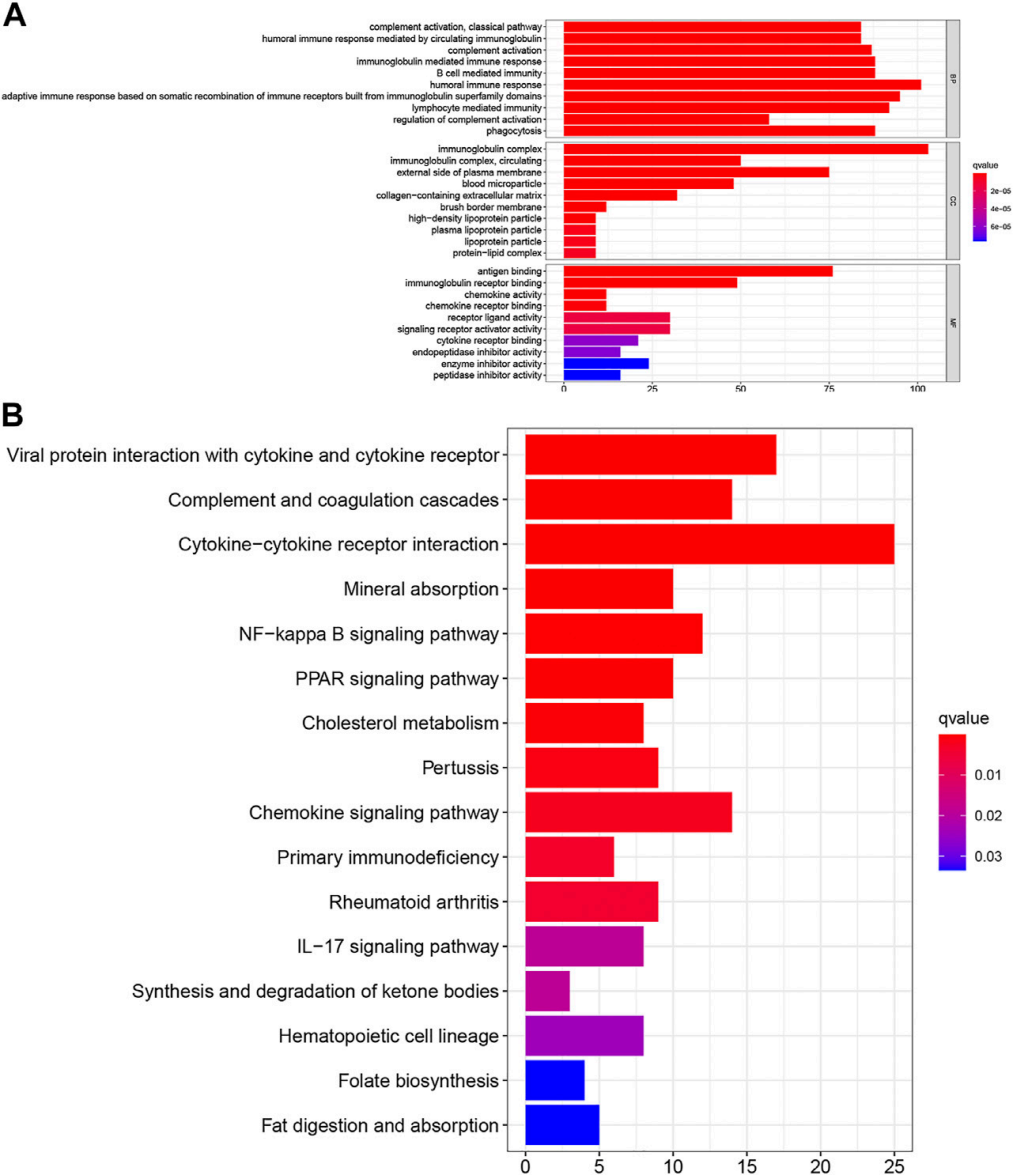
Investigation of the biological pathway about TNF-related signature. **(A)** The top 30 enriched GO analysis of the corresponding genes. **(B)** The top 16 enriched KEGG pathways of the corresponding genes.

To investigate further the potential functions of the TNF-related signature, we performed edgeR filtration (false discovery rate < 0.05, |log_2_FC > 1|) and identified 544 differentially expressed genes (DEGs), including 129 negatively related and 415 positively related genes (Supplementary Table S1). We applied GO enrichment analysis and KEGG pathway enrichment analysis. In the BP category, the TNF-related signature was highly enriched in humoral immune response, adaptive immune response based on somatic recombination of immune receptors built from immunoglobulin superfamily domains, and lymphocyte-mediated immunity (Figure 6A). In the CC category, the immunoglobulin complex and external side of the plasma membrane were markedly related to the signature. In the MF category, antigen binding, immunoglobulin receptor binding, receptor–ligand activity, and signaling receptor activator activity were highly enriched. KEGG analysis showed that the TNF-related signature was enriched in cytokine–cytokine receptor interaction, viral protein interaction with cytokines and cytokine receptors complement and coagulation cascades, and chemokine signaling pathway (Figure 6B).

### Immune Infiltration of the Tumor Necrosis Factor-Related Signature

**FIGURE 7:**
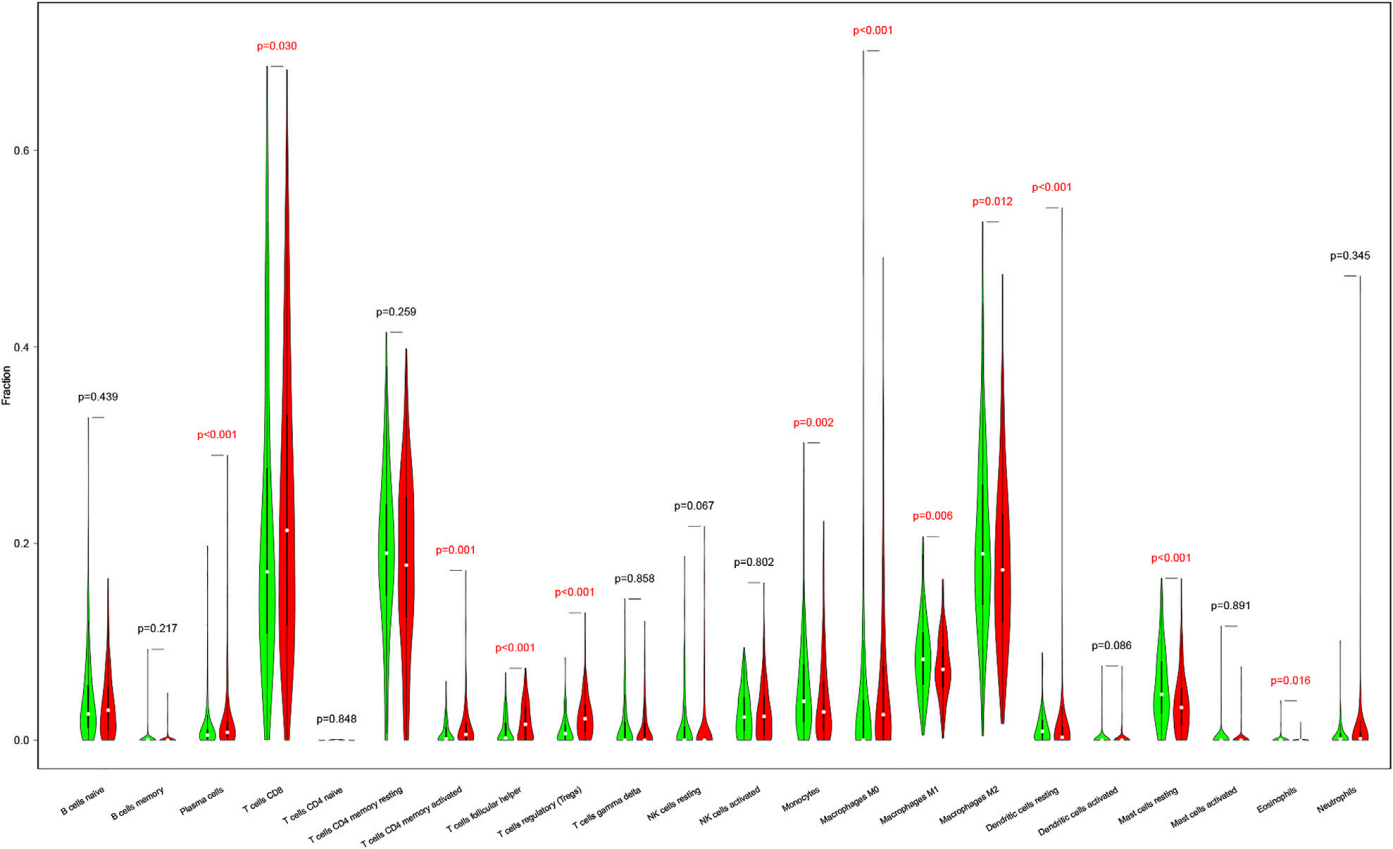
The differences in immune cell infiltration abundances between high- and low-risk patients. Red for the high-risk patients. Green for low-risk patients.

The results above illustrated that the TNF-related signature was significantly associated with immunity. To further explore the relationship between immune status and the signature, we quantified the immune infiltration between high- and low-risk groups through CIBERSORT. Supplementary Figure S2 showed the fractions of the 22 immune cells in every KIRC patient. Wilcoxon rank-sum test displayed remarkable discrepancy between the two groups. Plasma cells, CD8 T cells, CD4 memory activated T cells, follicular helper T cells, regulatory T cells (Tregs), and M0 were positively associated with the risk score, while monocytes, M1, M2, resting mast cells, and eosinophils were negatively associated with the risk score (Figure 7).

### Relationship Between Tumor Mutation Burden and Tumor Necrosis Factor-Related Signature

**FIGURE 8:**
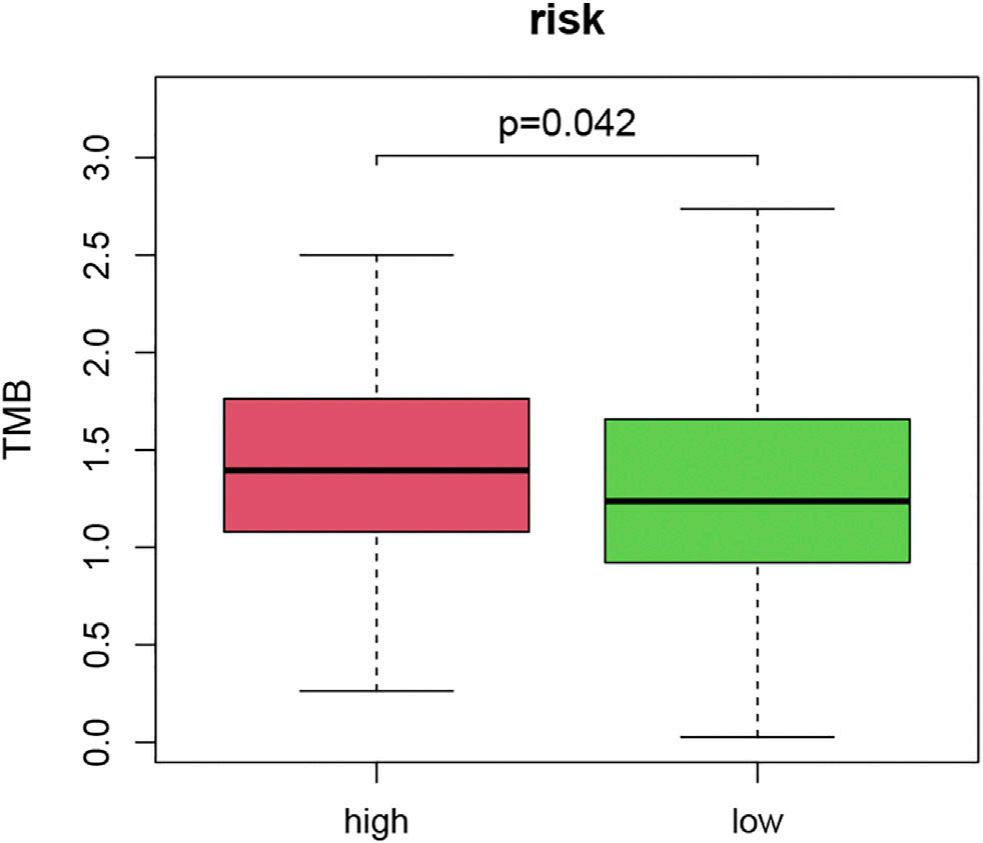
The relationship between TMB score and high- and low-risk patients.

TMB is a novel measurement to calculate the mutations of tumor cells. It can be a specific biomarker to assess the value of cancer immunotherapy (Merino et al., 2020). We applied the TNF-related signature to TMB calculation to explore their correlations. Mutation information of the top 30 most mutated genes was displayed in the waterfall, VHL and PBRM1 took up the majority of the mutation (Supplementary Figure S3). The mutations were classified into variant types, with missense mutations making up the majority, single nucleotide polymorphism was the most frequent type and C > T was the most common type of single nucleotide variant. We combined the risk score of patients with their TMB and compared the difference (Supplementary Figure S4). Patients with higher risk scores had a higher TMB (Figure 8). This meant that relevant immunotherapy might be applied to KIRC patients who were sensitive to the TNF-related signature.

## Discussion

Due to the comprehensive research of high-throughput sequencing and bioinformatics technology, more accurate and effective biomarkers have been used in cancer (Sharma, 2018). However, reliable biomarkers for risk assessment are still rare. Exploring new specific biomarkers for KIRC patients is of importance.

The TNF family regulates cell proliferation, migration and apoptosis. Stimulation or inhibition of the TNF superfamily signaling pathway may affect tumor progression. Therefore, we supposed that TNF family members might play an important role in predicting the prognosis of patients with KIRC (Vanamee and Faustman, 2018). We systematically studied the prognostic value of the TNF family genes in the KIRC cohort and found that most were protective factors. This finding was in line with previous studies (Dostert et al., 2019). We created a signature containing four genes (*TNFSF14*, *TNFRSF19*, *TNFRSF21* and *EDA*) to evaluate the prognosis of KIRC patients. *TNFSF14* can activate the immune cells, including T cells, dendritic cells and natural killer cells, by combining herpes virus entry mediator (HVEM) and lymphotoxin-β receptor (Mauri et al., 1998; Rooney et al., 2000; Granger et al., 2001). Previous research has found that *TNFSF14* is part of a cytokine network that participates in the innate and adaptive immune system for immune enhancement (Brunetti et al., 2020). *TNFRSF19* is expressed in the ureteral bud during embryonic development. It is present in the stem cells of adult kidneys to maintain homeostasis and regeneration and to regulate nuclear factor (NF)-κB activity by combining with β-catenin (Schön et al., 2014; Schutgens et al., 2017). *TNFRSF21*, also known as *DR6*, has been revealed to play a role in activating NF-κB and MAPK8/JNK and inducing apoptosis. It is an α-helical integral membrane receptor protein that inhibits the growth of blood vessels in tumor tissues (Pan et al., 1998). *EDA* regulates the structure and cell number during organ development. It regulates target genes by activating the downstream NF-κB pathway to suppress the proliferation of tumor cells (Sadier et al., 2014).

To explore the efficacy of the signature by combining the four genes above, the survival curve and ROC curve were utilized. The results demonstrated the good performance of the signature (*p* < 0.0001, ROC = 0.712). The ICGC cohort was used to validate the universality of the signature. The result indicated that the signature could be validated by different databases. When comparing our results to previous studies, it should be pointed out that our signature is more universal and efficient.

We discovered that the signature-related genes play critical roles in immunobiological pathways. We further revealed that patients in the high-risk group were in an immune-active state. The immune cells such as Tregs, CD8^+^ T cells and macrophages were highly expressed in the high-risk group. Tregs play important roles in immune tolerance and immune homeostasis (Takeuchi and Nishikawa, 2016). Previous studies have indicated that in a variety of cancers, such as colon, breast and pancreas cancer, increased Tregs are associated with poor prognosis (Zhuo et al., 2015; Wang et al., 2017). M0 promotes cell proliferation and invasion (Qian and Pollard, 2010), and increased macrophages are associated with poor prognosis in RCC (Hajiran et al., 2020). CD8^+^ T cells are considered to be the main antitumor cells and preferred targeted immune cells for treating cancer (Farhood et al., 2019). All these results were according to our expectation, indicating that our results proved the validity of the signature and provided a direction for further research, such as the possibility of immunotherapy for KIRC.

TMB has become an emerging biomarker of immunotherapy for many cancers (Chan et al., 2019). A few studies have reported that patients with higher TMB scores benefit more from immunotherapy. To further explore the correlation between the TNF-related signature and prognosis of immunotherapy, we analyzed the discrepancy in TMB score of the two groups. We discovered that the TMB score was significantly higher in the high-risk group, which made it possible to predict the efficacy of immunotherapy.

Combining the results of the TMB and immune infiltration, we found that high-risk patients had an elevated level of TMB, T cells, and B cells, which indicated that patients were in a state of immune activation. Due to the high intrinsic resistance to conventional chemo- and radiotherapies and the rapid development of resistance to targeted therapy, immune checkpoint inhibitors (ICIs) have been one of the few effective therapies for RCC. Previous studies have reported that higher TMB is closely related to better OS after ICI treatment (Valero et al., 2021). The new findings in our study indicated that the signature may be a predictive biomarker to predict the efficacy of immunotherapy.

This study had several limitations. All the samples used to establish and verify the signature were retrospective samples, therefore validation by prospective samples is necessary. Although our study found that the signature might be associated with immunotherapy, the efficacy of the signature cloud not been validated due to the lack of data, the potential mechanism and practical role in clinical practice need further exploration.

In summary, this is believed to be the first study of the TNF-family-based signature for KIRC and we demonstrated its value. It has the potential to become a powerful tool in guiding the immunotherapy of KIRC patients in clinical practice.

## Data Availability

Public datasets were analyzed in this study. This data can be found here: TCGA database *via* the GDC portal (https://portal.gdc.cancer.gov/). International Cancer Genome Consortium (ICGC) database (https://dcc.icgc.org/projects).

## Abbreviations

CI, confidence interval; DR6, death receptor 6; GO, gene ontology; HR, hazard ratios; HVEM, herpes virus entry mediator; ICGC, the International Cancer Genome Consortium; ICI, immune checkpoint inhibitors; JNK, c-Jun N-terminal kinase; KEGG, the Kyoto Encyclopedia of Genes and Genomes; KIRC, kidney renal clear cell carcinoma; LTβR, lymphotoxin-β receptor; MAPK, mitogen-activated protein kinase; NF-κB, nuclear factor kappa-B; NK cells, natural killer cells; OS, overall survival; RCC, renal cell carcinoma; ROC, receiver operating characteristic; TCGA, The Cancer Genome Atlas; TMB, tumor mutation burden; TNF, the tumor necrosis factor; TNFRSF, the tumor necrosis factor receptor superfamily; TNFSF, the tumor necrosis factor ligands superfamily.
