# Supplementary material for: Analyzing and Validating the Prognostic Value of a TNF-Related Signature in Kidney Renal Clear Cell Carcinoma
Source: Front Mol Biosci. 2021 May 28;8:689037. doi: 10.3389/fmolb.2021.689037 (PMC8194470; doi:10.3389/fmolb.2021.689037)
Supplement: Supplementary file 1 [file Table1.DOCX]

Table 1 Characteristics of patients with KIRC

| Characteristics | Variable | Total | Percentages |
| --- | --- | --- | --- |
| Age | ≦65 | 352 | 65.55% |
|  | >65 | 185 | 34.45% |
| Gender | Male | 346 | 64.43% |
|  | Female | 191 | 35.57% |
| Grade | Grade 1 | 14 | 2.61% |
|  | Grade 2 | 230 | 42.83% |
|  | Grade 3 | 207 | 38.55% |
|  | Grade 4 | 78 | 14.52% |
|  | G X | 5 | 0.93% |
|  | Unknown | 3 | 0.56% |
| Stage | Stage I | 269 | 50.09% |
|  | Stage II | 57 | 10.61% |
|  | Stage III | 125 | 23.28% |
|  | Stage IV | 83 | 15.46% |
|  | Unknown | 3 | 0.56% |
| T | T1 | 275 | 51.21% |
|  | T2 | 69 | 12.85% |
|  | T3 | 182 | 33.89% |
|  | T4 | 11 | 2.05% |
| N | N0 | 240 | 44.69% |
|  | N1 | 17 | 3.17% |
|  | NX | 280 | 52.14% |
| M | M0 | 426 | 79.33% |
|  | M1 | 79 | 14.71% |
|  | MX | 30 | 5.59% |
|  | Unknown | 2 | 0.37% |
| Survival rate | Survival | 367 | 68.34% |
|  | Dead | 170 | 31.66% |
