# Supplementary figures and images for: Analyzing and Validating the Prognostic Value of a TNF-Related Signature in Kidney Renal Clear Cell Carcinoma
Source: Front Mol Biosci. 2021 May 28;8:689037. doi: 10.3389/fmolb.2021.689037 (PMC8194470; doi:10.3389/fmolb.2021.689037)

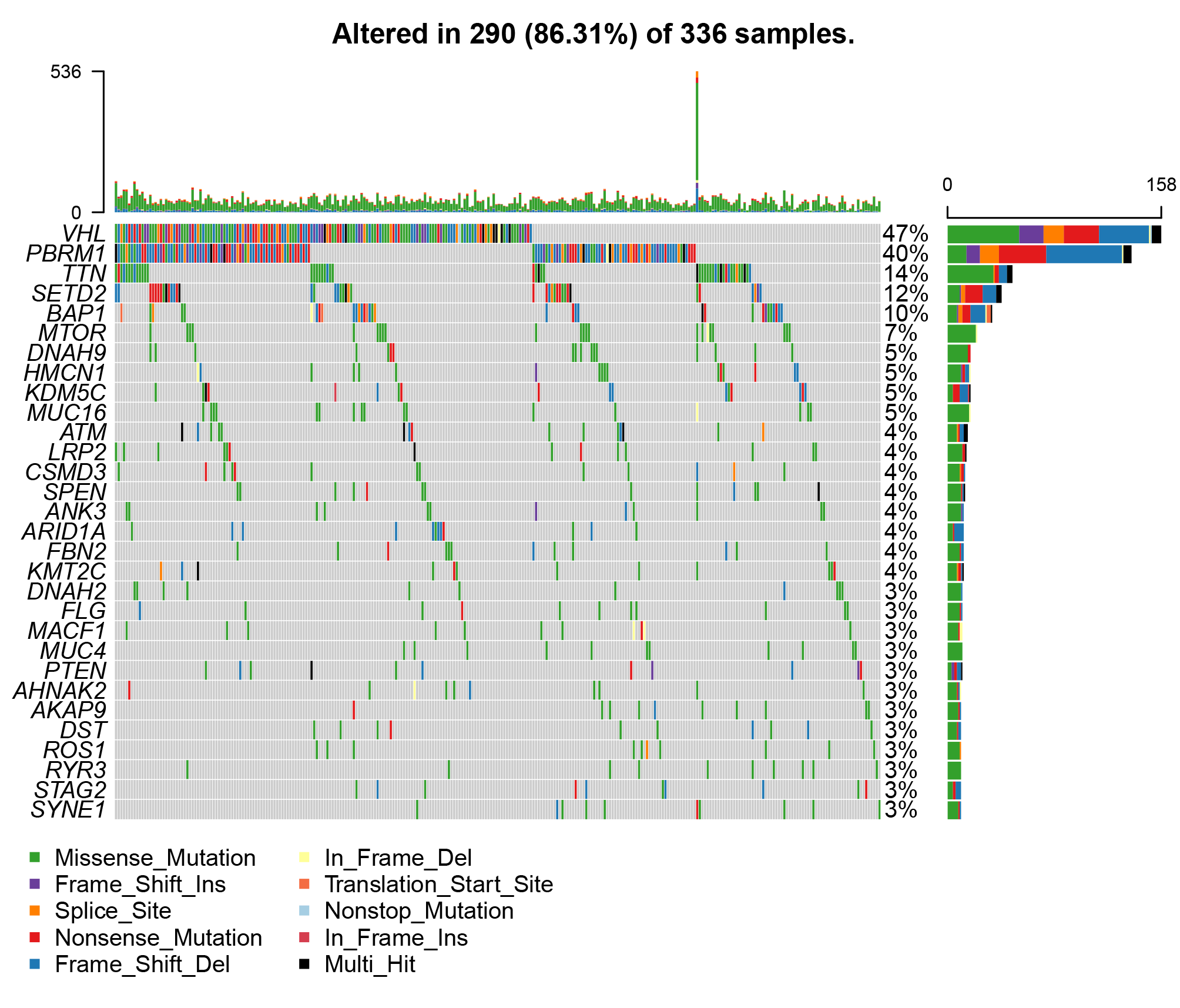

Supplement: Supplementary file 2 [file Image3.TIF]

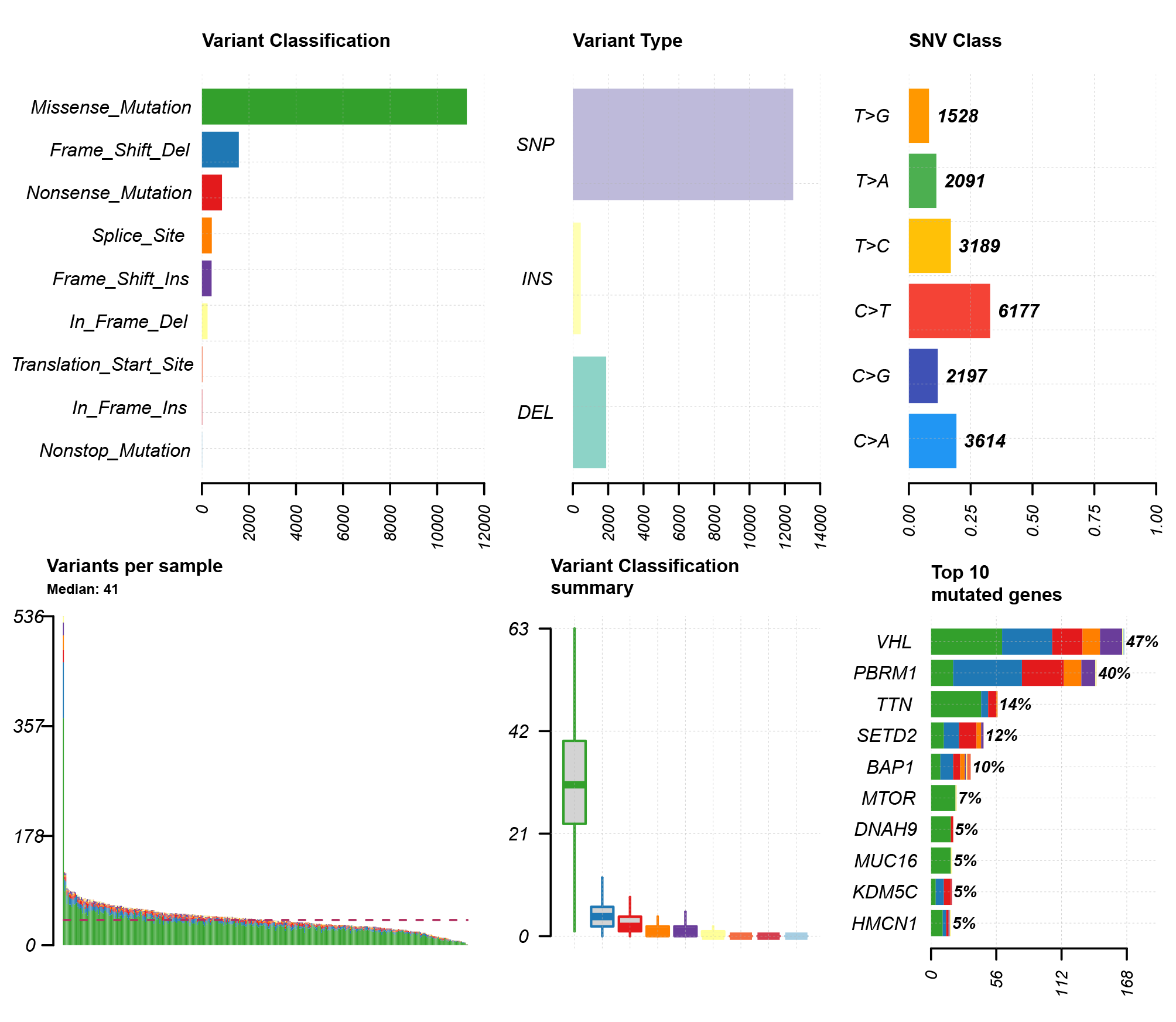

Supplement: Supplementary file 3 [file Image4.TIF]

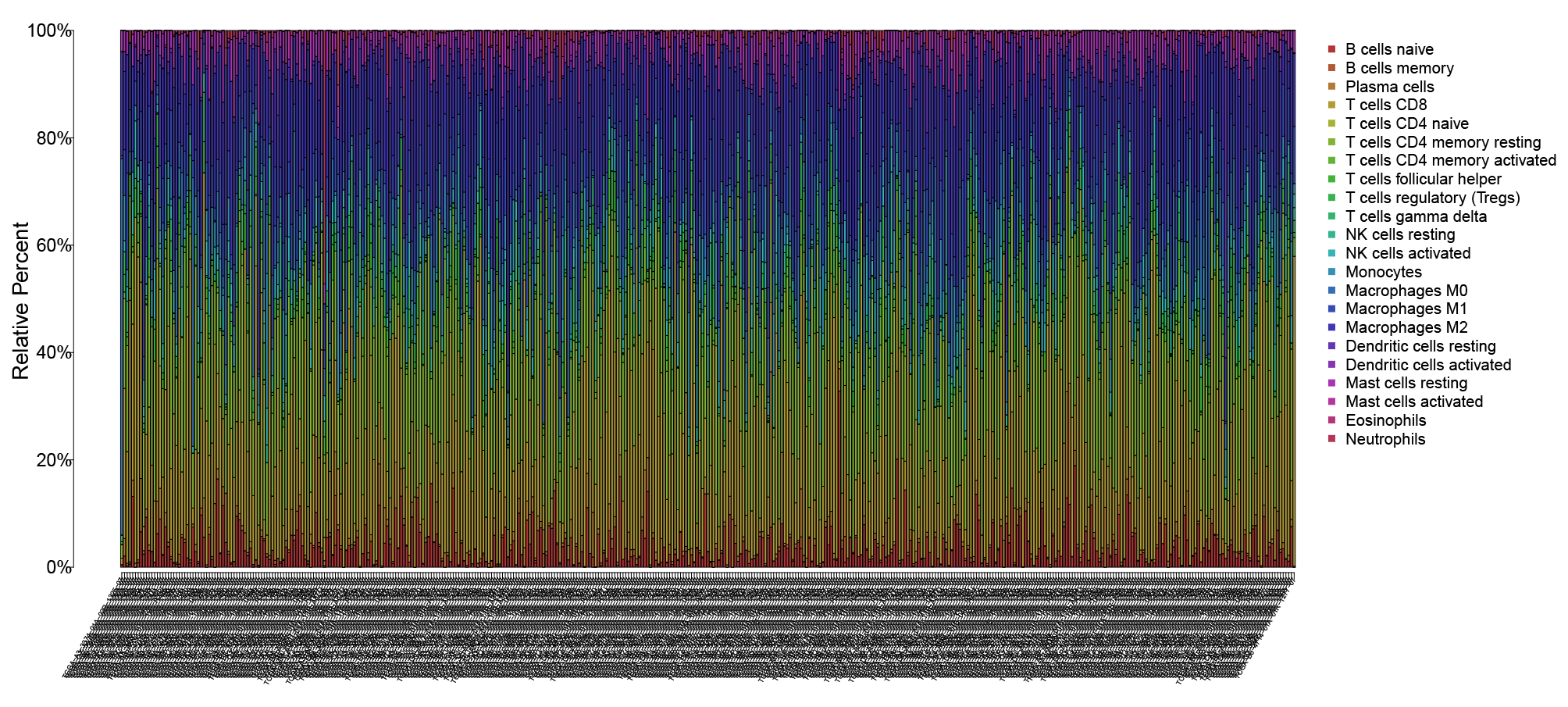

Supplement: Supplementary file 4 [file Image2.TIF]

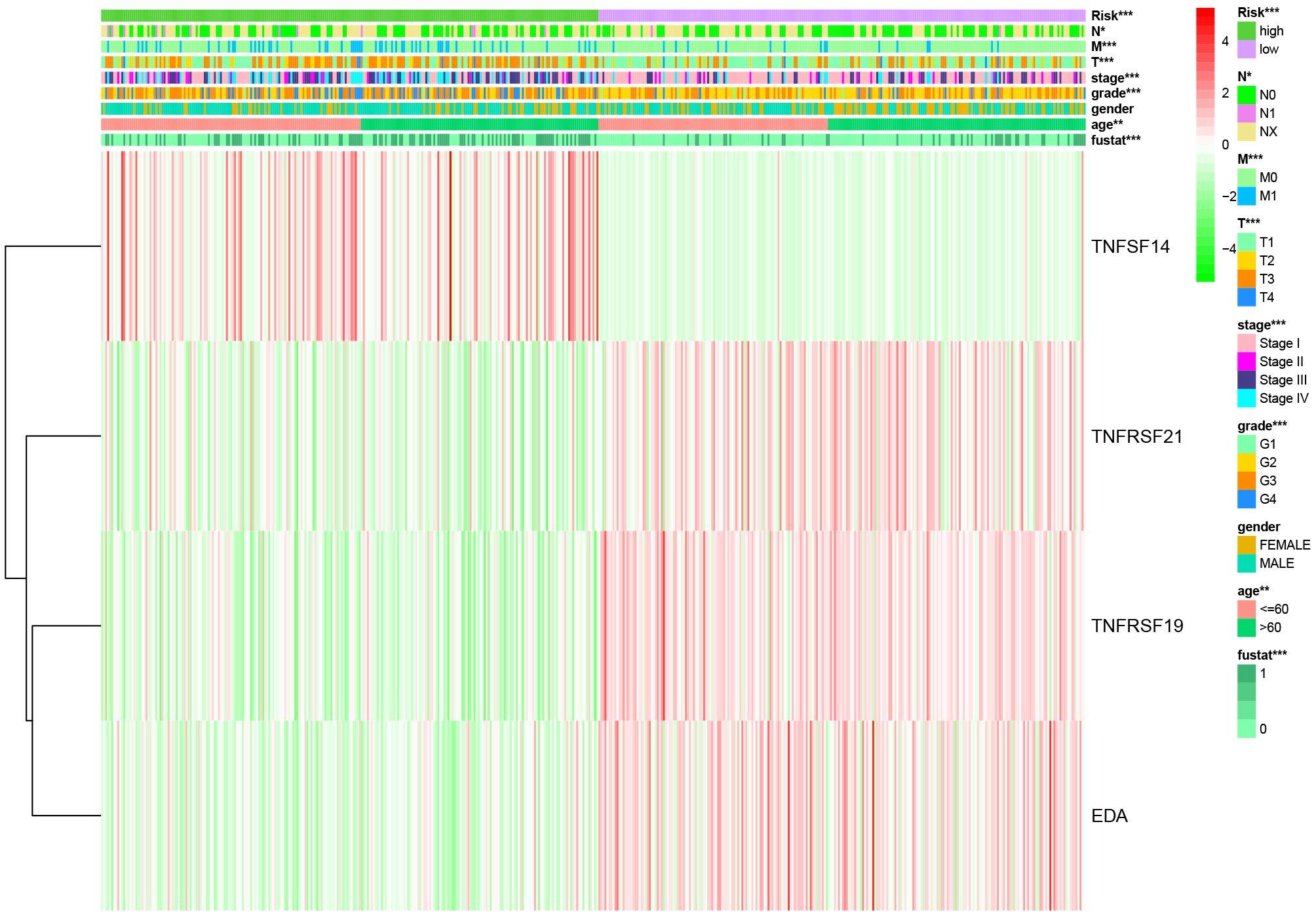

Supplement: Supplementary file 5 [file Image1.TIF]
