# Supplementary material for: Analyzing and Validating the Prognostic Value of a TNF-Related Signature in Kidney Renal Clear Cell Carcinoma
Source: Front Mol Biosci. 2021 May 28;8:689037. doi: 10.3389/fmolb.2021.689037 (PMC8194470; doi:10.3389/fmolb.2021.689037)
Supplement: Supplementary file 6 [file Table2.DOCX]

Table 2 15 genes associated with patients’ OS

| Gene | HR | Z | P value |
| --- | --- | --- | --- |
| CD27 | 1.125 | 1.832 | 0.067 |
| CD70 | 1.056 | 1.204 | 0.229 |
| EDA | 0.492 | -4.750 | <0.001 |
| EDA2R | 0.615 | -3.935 | <0.001 |
| FASLG | 1.229 | 1.922 | 0.055 |
| TNFRSF9 | 1.223 | 2.222 | 0.026 |
| TNFRSF11B | 0.838 | -2.400 | 0.016 |
| TNFRSF18 | 1.744 | 5.008 | <0.001 |
| TNFRSF19 | 0.588 | -5.906 | <0.001 |
| TNFRSF21 | 0.700 | -4.448 | <0.001 |
| TNFSF4 | 1.150 | 1.515 | 0.130 |
| TNFSF9 | 1.138 | 1.454 | 0.146 |
| TNFSF13 | 0.576 | -4.855 | <0.001 |
| TNFSF13B | 1.419 | 4.132 | <0.001 |
| TNFSF14 | 1.588 | 6.145 | <0.001 |

HR, hazard ratio; Z, Z test
