# Supplementary material for: Analyzing and Validating the Prognostic Value of a TNF-Related Signature in Kidney Renal Clear Cell Carcinoma
Source: Front Mol Biosci. 2021 May 28;8:689037. doi: 10.3389/fmolb.2021.689037 (PMC8194470; doi:10.3389/fmolb.2021.689037)
Supplement: Supplementary file 7 [file Table3.DOCX]

| gene | lowMean | highMean | logFC | pValue | fdr |
| --- | --- | --- | --- | --- | --- |
| IGKV4-1 | 34.01448517 | 153.2192882 | 2.171376766 | 1.14E-12 | 5.73E-12 |
| PRAME | 2.122944963 | 5.903830898 | 1.475584429 | 5.06E-23 | 1.37E-21 |
| IGLV4-60 | 1.754281887 | 13.4486943 | 2.938513621 | 2.64E-07 | 6.58E-07 |
| PLCG2 | 7.88205575 | 2.723846869 | -1.532926355 | 1.86E-05 | 3.68E-05 |
| IGHV3-74 | 8.03014355 | 27.24956282 | 1.762735401 | 4.89E-10 | 1.71E-09 |
| ESRRG | 1.644350879 | 0.643317212 | -1.353915987 | 1.50E-18 | 1.81E-17 |
| IGHV1-3 | 0.491031737 | 3.23428275 | 2.719557631 | 1.82E-07 | 4.62E-07 |
| TPSG1 | 1.045865652 | 5.084788899 | 2.281490339 | 1.01E-12 | 5.11E-12 |
| SLC27A2 | 22.48528342 | 8.898198669 | -1.337395854 | 1.88E-29 | 2.19E-27 |
| EDA | 2.130877671 | 1.052485184 | -1.01764785 | 3.40E-40 | 1.53E-36 |
| PPDPFL | 2.117961392 | 7.689739014 | 1.860258344 | 3.61E-16 | 2.99E-15 |
| TMEM171 | 11.80250075 | 5.655198579 | -1.061442985 | 5.11E-19 | 6.70E-18 |
| PANK1 | 4.043039228 | 1.888612813 | -1.098113238 | 4.35E-30 | 6.46E-28 |
| IGFLR1 | 0.712963647 | 1.593357267 | 1.160169365 | 2.12E-36 | 1.91E-33 |
| LINC01871 | 1.4968015 | 3.344404088 | 1.159866263 | 3.31E-30 | 5.27E-28 |
| IGHV4-31 | 3.792293444 | 28.55641344 | 2.912672287 | 2.96E-09 | 9.35E-09 |
| ACE2 | 30.49313728 | 14.78681052 | -1.044173689 | 4.90E-14 | 2.97E-13 |
| MT2P1 | 0.938915875 | 2.252875381 | 1.262699706 | 1.76E-10 | 6.53E-10 |
| FRAS1 | 3.418870824 | 1.663322753 | -1.039451776 | 5.01E-18 | 5.57E-17 |
| NPEPL1 | 1.989075135 | 4.429898618 | 1.155175908 | 4.41E-23 | 1.20E-21 |
| G6PC | 4.218001371 | 1.505497484 | -1.486319267 | 5.27E-18 | 5.84E-17 |
| IGKV3D-20 | 1.555927869 | 11.03427858 | 2.826145224 | 1.04E-10 | 3.99E-10 |
| IGKV2-29 | 1.08566382 | 4.909257362 | 2.176927365 | 6.46E-06 | 1.35E-05 |
| IGHV3-11 | 8.026474204 | 36.54200256 | 2.1867174 | 2.71E-08 | 7.60E-08 |
| IGKV3OR2-268 | 1.004940363 | 2.17395106 | 1.113209574 | 1.18E-10 | 4.53E-10 |
| SYCE1L | 1.109930275 | 2.361753768 | 1.089389509 | 9.08E-19 | 1.14E-17 |
| FLRT3 | 9.70787808 | 4.756746898 | -1.029180727 | 7.97E-16 | 6.25E-15 |
| CHI3L2 | 1.161166861 | 2.945566751 | 1.342969943 | 1.30E-19 | 1.84E-18 |
| FOXP3 | 0.63037419 | 1.731291627 | 1.457568389 | 6.55E-33 | 2.46E-30 |
| DNAJB13 | 0.72310554 | 1.610375633 | 1.155119113 | 1.85E-17 | 1.88E-16 |
| TRIM54 | 0.746656515 | 3.382291962 | 2.179484584 | 6.54E-20 | 9.67E-19 |
| MYO7B | 3.541306398 | 1.720615657 | -1.041356802 | 0.000106613 | 0.00019161 |
| CD79A | 1.643964391 | 7.389600928 | 2.168317404 | 9.27E-19 | 1.16E-17 |
| F3 | 2.289263449 | 5.7480383 | 1.328186177 | 0.000191898 | 0.000334228 |
| SPINK13 | 3.271507324 | 8.40904142 | 1.361985851 | 9.03E-19 | 1.13E-17 |
| FGB | 36.99718037 | 99.47302598 | 1.42689004 | 5.41E-11 | 2.15E-10 |
| ADA | 3.139409362 | 6.432905428 | 1.034977318 | 2.30E-28 | 2.24E-26 |
| ADAM8 | 1.506231336 | 3.655978822 | 1.279314348 | 2.32E-27 | 1.69E-25 |
| PSTPIP1 | 1.193633913 | 2.545142457 | 1.092385979 | 1.58E-26 | 9.19E-25 |
| AKR1B10 | 4.350891201 | 11.12692141 | 1.354671638 | 4.65E-16 | 3.75E-15 |
| C10orf99 | 6.730239769 | 35.51554231 | 2.399720707 | 4.31E-17 | 4.13E-16 |
| IGKV2-24 | 5.126518573 | 26.87308506 | 2.390110627 | 6.43E-09 | 1.95E-08 |
| TRIB3 | 11.13935948 | 25.47665696 | 1.1935097 | 1.02E-20 | 1.73E-19 |
| IGHA1 | 157.8303766 | 622.2016584 | 1.979007342 | 1.63E-14 | 1.06E-13 |
| AP000757.1 | 1.655736034 | 0.608872069 | -1.44326165 | 2.83E-05 | 5.46E-05 |
| HPD | 2.525333547 | 5.495607566 | 1.121805035 | 0.001137242 | 0.001792604 |
| IGHV1-69 | 2.943008843 | 15.88984324 | 2.43274111 | 9.40E-11 | 3.63E-10 |
| IGKV6-21 | 0.890272085 | 6.320401727 | 2.827698035 | 2.83E-09 | 8.97E-09 |
| CYP8B1 | 1.3160688 | 2.729383089 | 1.052339991 | 0.000889854 | 0.001423387 |
| PCK1 | 32.48405327 | 12.31091986 | -1.399793096 | 2.49E-16 | 2.10E-15 |
| CTXN3 | 1.765070041 | 0.832718393 | -1.083824838 | 0.000200602 | 0.000348222 |
| F2 | 0.394256523 | 4.674861761 | 3.567717175 | 4.98E-25 | 1.96E-23 |
| IGLV3-21 | 18.13360939 | 96.58588533 | 2.413146261 | 2.56E-12 | 1.23E-11 |
| SFRP1 | 3.659618243 | 1.687000631 | -1.117232647 | 0.001765958 | 0.002714667 |
| IGKV3D-15 | 0.998583929 | 3.292224129 | 1.721106962 | 2.93E-09 | 9.27E-09 |
| IGHGP | 6.544801325 | 40.21262715 | 2.619227289 | 4.99E-16 | 4.02E-15 |
| IGFBP1 | 8.076345257 | 34.27712628 | 2.085471671 | 1.80E-10 | 6.69E-10 |
| KIT | 9.264479783 | 2.570496499 | -1.849662922 | 5.78E-06 | 1.22E-05 |
| CYP4A11 | 31.64786858 | 10.68408102 | -1.566645518 | 1.32E-12 | 6.57E-12 |
| CYP4F3 | 0.640742105 | 1.862840354 | 1.539688337 | 4.75E-07 | 1.15E-06 |
| C5orf46 | 6.508827198 | 20.93649732 | 1.685550581 | 1.60E-16 | 1.40E-15 |
| CXCL13 | 2.137054788 | 8.086045002 | 1.919810338 | 3.19E-24 | 1.06E-22 |
| TMEM246-AS1 | 1.469939635 | 0.666606488 | -1.140849647 | 1.93E-09 | 6.25E-09 |
| IGHG1 | 86.35404094 | 652.8228914 | 2.918356051 | 1.56E-16 | 1.37E-15 |
| TSPAN5 | 4.172978777 | 1.927490329 | -1.114353962 | 3.84E-05 | 7.28E-05 |
| GHR | 4.371033825 | 2.035114807 | -1.102864359 | 7.25E-21 | 1.28E-19 |
| CD3D | 7.934942025 | 16.3826973 | 1.045881319 | 1.36E-22 | 3.38E-21 |
| IGLV8-61 | 7.131333543 | 33.91437149 | 2.249652969 | 7.12E-12 | 3.21E-11 |
| BEST4 | 0.783453335 | 1.951780908 | 1.316871866 | 2.86E-12 | 1.37E-11 |
| SLC5A8 | 13.41206845 | 6.157152998 | -1.123196428 | 1.84E-16 | 1.60E-15 |
| CYP17A1 | 6.665581139 | 1.09208321 | -2.609647878 | 4.21E-06 | 9.03E-06 |
| BATF | 2.260409942 | 5.473250294 | 1.275813393 | 1.33E-31 | 2.95E-29 |
| IGLV9-49 | 1.846148043 | 48.62823671 | 4.719204127 | 5.05E-11 | 2.01E-10 |
| LINC01426 | 2.656302725 | 5.726971922 | 1.108352958 | 5.35E-17 | 5.08E-16 |
| IGKV5-2 | 0.733306135 | 10.14867212 | 3.790731554 | 9.13E-07 | 2.12E-06 |
| ANGPTL8 | 0.235227753 | 6.032926298 | 4.680727769 | 8.33E-23 | 2.19E-21 |
| PPP1R1A | 2.613260828 | 11.68380821 | 2.16058755 | 7.80E-22 | 1.69E-20 |
| WDR72 | 10.09350758 | 4.144453112 | -1.284173964 | 7.21E-35 | 4.64E-32 |
| IGLV2-18 | 1.769440092 | 8.866552625 | 2.325080367 | 3.32E-10 | 1.18E-09 |
| PRIMA1 | 6.275683857 | 14.96747612 | 1.253986386 | 2.47E-16 | 2.09E-15 |
| COMP | 1.242879661 | 2.780606497 | 1.161712975 | 5.15E-07 | 1.24E-06 |
| IGKV1D-16 | 0.798897643 | 2.835661452 | 1.827602722 | 2.29E-10 | 8.38E-10 |
| IGLV1-47 | 19.91605437 | 53.7638938 | 1.432705768 | 6.66E-12 | 3.02E-11 |
| THSD7A | 3.937805007 | 1.890066751 | -1.058954487 | 1.76E-14 | 1.14E-13 |
| MAP7 | 14.23581877 | 6.625023572 | -1.103527979 | 1.58E-33 | 7.36E-31 |
| IGHV4-59 | 9.97071042 | 55.0021055 | 2.46371864 | 1.91E-11 | 8.04E-11 |
| NUPR2 | 1.96504814 | 0.683233598 | -1.52411383 | 0.009281004 | 0.012924417 |
| IL2RG | 9.343269461 | 18.72637682 | 1.003072413 | 9.55E-23 | 2.49E-21 |
| IGLV7-46 | 4.424486219 | 58.98842051 | 3.736849939 | 8.92E-12 | 3.96E-11 |
| IGHV1-18 | 15.23088985 | 98.86462675 | 2.698454192 | 8.93E-10 | 3.01E-09 |
| MYL3 | 5.630969356 | 2.200528796 | -1.355533049 | 7.51E-21 | 1.31E-19 |
| KLHDC7B | 0.682008142 | 1.480806896 | 1.118522652 | 3.11E-17 | 3.03E-16 |
| MMP12 | 0.705167097 | 1.583531158 | 1.16710819 | 5.89E-18 | 6.48E-17 |
| SOWAHB | 3.878333437 | 1.698698342 | -1.191007165 | 7.06E-37 | 7.35E-34 |
| HSPA7 | 2.919988149 | 6.270661879 | 1.102655216 | 5.81E-13 | 3.03E-12 |
| SNORD14E | 1.357139066 | 5.273413269 | 1.958168502 | 1.32E-15 | 1.00E-14 |
| PKP3 | 0.681651262 | 1.816759895 | 1.414262026 | 1.42E-11 | 6.11E-11 |
| C1orf210 | 9.400916839 | 4.35784657 | -1.109186062 | 7.59E-26 | 3.59E-24 |
| EGF | 2.010181954 | 0.937579209 | -1.100313611 | 0.001205889 | 0.001896612 |
| ORM2 | 0.1019501 | 5.659383785 | 5.794709881 | 1.25E-09 | 4.14E-09 |
| AC105446.1 | 0.806724265 | 2.206007889 | 1.451290396 | 2.22E-20 | 3.55E-19 |
| IGLV2-14 | 30.17842636 | 170.9938166 | 2.502354671 | 1.48E-11 | 6.34E-11 |
| CPN2 | 1.494243973 | 3.942417807 | 1.399664954 | 0.000389176 | 0.000653344 |
| AC008760.2 | 3.848504473 | 8.755238328 | 1.185848527 | 5.01E-11 | 2.00E-10 |
| IGKV3-11 | 25.24333674 | 136.4288867 | 2.434174618 | 1.06E-14 | 7.05E-14 |
| TMEM38A | 4.987411172 | 1.697269061 | -1.555075859 | 2.79E-20 | 4.37E-19 |
| CXCL6 | 2.916270945 | 6.308062664 | 1.113072228 | 1.96E-05 | 3.85E-05 |
| FABP6 | 12.00561914 | 27.33403785 | 1.186988787 | 2.79E-19 | 3.80E-18 |
| ENAM | 1.817996869 | 0.607634991 | -1.58107286 | 2.46E-30 | 4.16E-28 |
| NPR3 | 38.12852819 | 14.45587366 | -1.399215039 | 4.01E-25 | 1.63E-23 |
| U62317.1 | 0.616404265 | 1.564902095 | 1.34412365 | 1.55E-27 | 1.18E-25 |
| SLC22A6 | 18.54643921 | 6.903948527 | -1.425648612 | 1.12E-08 | 3.30E-08 |
| LINC01559 | 1.10220184 | 3.52260512 | 1.676254318 | 1.47E-12 | 7.28E-12 |
| SNORD94 | 2.532014427 | 7.077862715 | 1.483028154 | 2.43E-06 | 5.37E-06 |
| IGHJ2 | 1.905519838 | 8.624382156 | 2.178236479 | 1.83E-10 | 6.80E-10 |
| RARRES1 | 3.170518819 | 10.04446167 | 1.663609399 | 2.05E-24 | 7.04E-23 |
| BASP1 | 6.136455693 | 14.00800399 | 1.190773874 | 9.96E-28 | 8.02E-26 |
| IRF6 | 7.094019733 | 3.265781343 | -1.119175144 | 1.11E-15 | 8.51E-15 |
| IGKV1D-13 | 0.562146213 | 2.447941937 | 2.122552012 | 1.41E-07 | 3.63E-07 |
| APCS | 0.166674132 | 3.972376559 | 4.57490027 | 0.009079259 | 0.012664335 |
| APOL1 | 63.51498421 | 152.5260971 | 1.263887216 | 8.75E-16 | 6.81E-15 |
| IGLV7-43 | 4.250174072 | 13.48382287 | 1.665635745 | 5.62E-12 | 2.57E-11 |
| TRBV28 | 5.30490081 | 12.06001022 | 1.184833449 | 2.17E-25 | 9.14E-24 |
| IGHV4-55 | 0.695090531 | 3.026963293 | 2.122598384 | 2.87E-10 | 1.03E-09 |
| IGKJ5 | 1.424410316 | 6.712414129 | 2.236466937 | 6.95E-14 | 4.11E-13 |
| AC103702.2 | 1.13857154 | 3.021073255 | 1.407836223 | 3.16E-13 | 1.71E-12 |
| MELTF-AS1 | 0.949138254 | 1.941794271 | 1.032700204 | 9.62E-14 | 5.59E-13 |
| VSTM2L | 0.63566776 | 1.833552569 | 1.528296805 | 7.10E-13 | 3.67E-12 |
| RTN4RL1 | 1.029151646 | 2.153443149 | 1.065189657 | 7.52E-10 | 2.56E-09 |
| CRYBB3 | 0.898762064 | 2.197361908 | 1.289761367 | 0.000673065 | 0.001094216 |
| MAT1A | 0.488152779 | 1.743475608 | 1.83656153 | 9.51E-21 | 1.63E-19 |
| PGF | 18.5713393 | 39.88713069 | 1.102845485 | 4.27E-09 | 1.32E-08 |
| TF | 0.505750581 | 8.369625149 | 4.048665033 | 1.68E-15 | 1.25E-14 |
| BCL2A1 | 2.746286202 | 5.779925326 | 1.073568871 | 3.07E-19 | 4.14E-18 |
| LTB | 3.656130674 | 9.007772191 | 1.300852707 | 1.27E-29 | 1.56E-27 |
| HIST1H2BG | 1.368116946 | 2.781676224 | 1.02376295 | 1.74E-10 | 6.48E-10 |
| TMEM213 | 14.37203687 | 0.881935475 | -4.026447623 | 0.003062575 | 0.00455213 |
| LINC01358 | 1.958292021 | 4.418855682 | 1.174076898 | 6.56E-16 | 5.19E-15 |
| DES | 4.368974961 | 10.00564819 | 1.195447889 | 0.001386274 | 0.002161718 |
| SST | 91.79564721 | 19.60964044 | -2.226862662 | 5.61E-08 | 1.52E-07 |
| IGLV3-9 | 2.520198244 | 14.5181632 | 2.526249811 | 4.82E-13 | 2.55E-12 |
| AC133644.2 | 0.771460912 | 2.376699999 | 1.623294844 | 1.05E-23 | 3.22E-22 |
| PAIP2B | 3.925930672 | 1.598520946 | -1.296297048 | 2.88E-33 | 1.22E-30 |
| IGLV3-19 | 28.89125193 | 223.8092666 | 2.953565145 | 9.48E-11 | 3.66E-10 |
| SLC6A19 | 18.03205105 | 3.131283716 | -2.525737367 | 2.07E-20 | 3.32E-19 |
| IGKV6D-21 | 1.002218787 | 2.716879021 | 1.438752839 | 2.98E-09 | 9.40E-09 |
| ZBTB7C | 0.653131355 | 1.53276119 | 1.230687863 | 3.08E-08 | 8.59E-08 |
| NKAIN4 | 3.139805226 | 6.439262842 | 1.036220474 | 5.63E-07 | 1.35E-06 |
| ATP2B2 | 2.23166349 | 1.110304747 | -1.007163791 | 1.69E-12 | 8.35E-12 |
| IGHV2-5 | 1.461725042 | 5.516165576 | 1.915993804 | 1.92E-07 | 4.86E-07 |
| CLMP | 0.865667121 | 1.785952564 | 1.044809491 | 1.08E-09 | 3.62E-09 |
| MISP | 0.973439112 | 2.651812781 | 1.445816277 | 2.04E-08 | 5.81E-08 |
| CRP | 0.317636909 | 8.066908207 | 4.666565371 | 6.70E-14 | 3.97E-13 |
| IGHV4-4 | 1.013695167 | 4.684032498 | 2.208127208 | 5.85E-05 | 0.000108515 |
| CD7 | 1.960824965 | 6.07326616 | 1.631011839 | 2.24E-25 | 9.39E-24 |
| SLC18A3 | 0.618504017 | 2.204662809 | 1.83370315 | 6.68E-07 | 1.58E-06 |
| OGDHL | 22.95104169 | 8.765667391 | -1.388623793 | 2.75E-15 | 1.99E-14 |
| ZAP70 | 1.224067142 | 2.493328577 | 1.026390322 | 1.43E-19 | 2.02E-18 |
| MT2A | 89.84351996 | 194.4317275 | 1.113777301 | 2.10E-16 | 1.80E-15 |
| CHRDL2 | 0.313054146 | 1.725643441 | 2.462650288 | 8.38E-17 | 7.64E-16 |
| IGKV2-30 | 0.869854733 | 3.142804858 | 1.853206301 | 2.73E-11 | 1.12E-10 |
| SNORA33 | 2.246345573 | 4.823589543 | 1.102527263 | 2.14E-17 | 2.15E-16 |
| MIR155HG | 1.608550358 | 3.742792716 | 1.218354049 | 2.92E-21 | 5.71E-20 |
| PCED1B-AS1 | 2.074838785 | 4.18773757 | 1.013171793 | 3.65E-32 | 9.67E-30 |
| IGLV10-54 | 3.31424879 | 11.48412106 | 1.792886634 | 7.71E-08 | 2.06E-07 |
| SLC6A18 | 8.436540624 | 2.81570464 | -1.583155541 | 1.87E-08 | 5.37E-08 |
| LGALS12 | 1.390812513 | 3.362326577 | 1.273531906 | 1.08E-12 | 5.45E-12 |
| SLC34A1 | 3.056586143 | 0.932231211 | -1.713161507 | 7.67E-05 | 0.000140292 |
| HP | 0.315363651 | 45.07233893 | 7.159082124 | 1.04E-25 | 4.68E-24 |
| INHBE | 0.545383839 | 1.85766587 | 1.768147178 | 8.81E-13 | 4.51E-12 |
| CRABP1 | 0.416569215 | 6.583125626 | 3.982144597 | 5.00E-06 | 1.06E-05 |
| LINC01554 | 2.568472515 | 7.613832376 | 1.567712173 | 2.86E-09 | 9.05E-09 |
| IGKV1-9 | 9.602166812 | 60.78501865 | 2.66228389 | 8.60E-11 | 3.34E-10 |
| IGHA2 | 23.33044426 | 94.38522921 | 2.016347324 | 1.10E-12 | 5.55E-12 |
| SCNN1G | 0.698930022 | 3.828246483 | 2.453463798 | 0.002762187 | 0.004131063 |
| DPEP1 | 6.328583423 | 2.35611466 | -1.425472857 | 0.016050822 | 0.021662684 |
| ANKRD2 | 0.647961288 | 1.524655979 | 1.234504225 | 0.000172306 | 0.000301659 |
| AP000439.2 | 16.92457651 | 6.71856886 | -1.332893876 | 6.38E-11 | 2.51E-10 |
| PIM2 | 7.018145911 | 15.84856321 | 1.175190208 | 4.19E-23 | 1.15E-21 |
| AC103563.1 | 0.3716244 | 2.080186788 | 2.484795947 | 3.54E-06 | 7.68E-06 |
| MT1A | 2.544415141 | 5.891341054 | 1.211261998 | 5.73E-08 | 1.55E-07 |
| IGHV2-70D | 0.911925254 | 5.479447834 | 2.587043036 | 1.11E-07 | 2.91E-07 |
| IGKV1-5 | 22.12674064 | 131.0629718 | 2.566397292 | 2.82E-13 | 1.54E-12 |
| MIR5690 | 0.642673972 | 1.66169362 | 1.370495455 | 0.003437869 | 0.005069292 |
| SLC3A1 | 89.57429754 | 44.22594613 | -1.018191817 | 4.35E-30 | 6.46E-28 |
| USP2 | 8.305169553 | 3.680737807 | -1.174014637 | 2.14E-14 | 1.36E-13 |
| IGHV3-49 | 4.629861807 | 30.73894766 | 2.731026738 | 7.41E-09 | 2.23E-08 |
| ADAM12 | 1.130610769 | 2.282734891 | 1.013660976 | 3.72E-17 | 3.61E-16 |
| CA4 | 5.751469148 | 2.35191905 | -1.290092118 | 5.32E-18 | 5.90E-17 |
| AL627309.7 | 0.789664794 | 1.719227895 | 1.12244852 | 5.47E-12 | 2.51E-11 |
| AC011352.1 | 1.005309621 | 2.895405966 | 1.526125744 | 2.86E-15 | 2.07E-14 |
| SCARNA6 | 1.277702179 | 12.55016273 | 3.296082569 | 0.000209989 | 0.00036363 |
| PAH | 7.983118875 | 2.562515347 | -1.639391849 | 8.84E-05 | 0.00016051 |
| PDK4 | 234.7555466 | 103.5835781 | -1.180363945 | 1.47E-14 | 9.63E-14 |
| IGHV3-33 | 5.721623579 | 35.11131671 | 2.617439608 | 8.98E-12 | 3.98E-11 |
| C11orf86 | 0.45725411 | 3.344350527 | 2.870658022 | 1.02E-12 | 5.15E-12 |
| CD72 | 1.558359354 | 3.290458676 | 1.07826075 | 6.30E-24 | 1.99E-22 |
| MIR8071-2 | 0.592418775 | 1.94238464 | 1.713139651 | 5.07E-13 | 2.67E-12 |
| IGHV2-26 | 2.849134027 | 12.76128919 | 2.163178688 | 2.35E-09 | 7.52E-09 |
| IGLV3-27 | 2.006045473 | 6.626332688 | 1.723856329 | 3.13E-10 | 1.12E-09 |
| RNU4-2 | 32.80229022 | 129.3710752 | 1.979646645 | 0.00340248 | 0.005022037 |
| TMEM174 | 14.03823329 | 5.101754167 | -1.460296095 | 8.19E-17 | 7.50E-16 |
| XCL1 | 0.650008479 | 1.374703451 | 1.080589994 | 2.04E-21 | 4.09E-20 |
| LAG3 | 2.200333767 | 5.454087605 | 1.309615491 | 2.60E-21 | 5.14E-20 |
| C4BPA | 0.142246246 | 1.879181968 | 3.723642289 | 5.57E-10 | 1.93E-09 |
| IGHV1-45 | 0.374764199 | 2.028626492 | 2.436448217 | 7.13E-09 | 2.15E-08 |
| DERL3 | 1.474341708 | 4.536790985 | 1.621601259 | 6.45E-25 | 2.46E-23 |
| APOC3 | 0.252705189 | 19.986028 | 6.30539268 | 0.022523026 | 0.029672393 |
| MIR4768 | 3.93589275 | 8.161503845 | 1.0521441 | 1.06E-08 | 3.14E-08 |
| IGLV3-10 | 8.759938073 | 65.11487006 | 2.893994468 | 4.75E-12 | 2.20E-11 |
| MMP1 | 3.557745551 | 8.825690081 | 1.310745754 | 0.023864417 | 0.031284189 |
| CARD11 | 2.410670869 | 5.738462108 | 1.251229459 | 4.96E-24 | 1.59E-22 |
| TIGIT | 0.786637168 | 1.668262518 | 1.084576069 | 2.95E-21 | 5.76E-20 |
| SNORA74B | 1.47333837 | 5.940868028 | 2.01158494 | 9.34E-05 | 0.000169217 |
| SLC22A11 | 22.36561274 | 10.80327472 | -1.049813591 | 7.31E-17 | 6.77E-16 |
| CUBN | 46.08975883 | 22.20731026 | -1.053411554 | 9.19E-17 | 8.30E-16 |
| IGHV3-21 | 11.46921078 | 52.04761719 | 2.182065997 | 2.40E-11 | 9.96E-11 |
| IGLV1-40 | 20.43066699 | 344.3511784 | 4.075072405 | 4.55E-12 | 2.11E-11 |
| AC092336.1 | 3.503873091 | 7.705132913 | 1.136869322 | 1.27E-05 | 2.55E-05 |
| GBP5 | 3.025814169 | 6.392290412 | 1.079009567 | 7.74E-14 | 4.56E-13 |
| CXCL8 | 2.971871517 | 7.525927292 | 1.340497607 | 3.24E-07 | 7.99E-07 |
| AC073218.2 | 1.885012409 | 5.279657418 | 1.4858703 | 5.27E-19 | 6.89E-18 |
| IGKV3D-11 | 0.690717134 | 3.233164708 | 2.226780088 | 2.27E-11 | 9.47E-11 |
| SERPINC1 | 0.179189636 | 5.324205354 | 4.893007115 | 0.000566955 | 0.000930391 |
| PADI3 | 0.23216104 | 1.900198059 | 3.032952007 | 2.38E-17 | 2.38E-16 |
| SCARNA10 | 2.811462019 | 11.86416363 | 2.077217938 | 0.000159765 | 0.00028072 |
| IGLV2-8 | 7.608773522 | 27.72905918 | 1.865662842 | 1.86E-11 | 7.87E-11 |
| AC084117.1 | 1.416045332 | 2.90264539 | 1.03550088 | 8.96E-15 | 6.03E-14 |
| COLCA1 | 1.376773319 | 0.636710026 | -1.112582657 | 1.19E-18 | 1.46E-17 |
| FGA | 6.518312224 | 47.25514211 | 2.857900963 | 2.42E-16 | 2.05E-15 |
| FOXI2 | 2.742503146 | 0.10923362 | -4.65000441 | 1.05E-13 | 6.04E-13 |
| FGG | 13.47656484 | 51.4346329 | 1.932287306 | 1.10E-11 | 4.81E-11 |
| AC092611.2 | 2.039296152 | 0.99439285 | -1.036183475 | 1.63E-29 | 1.92E-27 |
| KRT223P | 4.552359612 | 14.0477839 | 1.625656125 | 6.40E-11 | 2.52E-10 |
| NCR3LG1 | 2.512017508 | 1.0794595 | -1.218537403 | 3.32E-27 | 2.30E-25 |
| IGHV3-73 | 4.104670511 | 38.11693133 | 3.215093653 | 1.54E-07 | 3.94E-07 |
| IGHG3 | 63.53703613 | 184.5479461 | 1.538325984 | 5.95E-18 | 6.54E-17 |
| PTGER3 | 11.67619186 | 5.002307657 | -1.222904126 | 4.82E-13 | 2.55E-12 |
| IGLC2 | 63.34409573 | 402.3880667 | 2.667305463 | 1.35E-18 | 1.63E-17 |
| CD27 | 4.693371895 | 10.18447065 | 1.11767431 | 1.11E-20 | 1.88E-19 |
| HAO2 | 17.48920262 | 7.608237165 | -1.20083039 | 9.59E-15 | 6.42E-14 |
| C1S | 30.80391204 | 90.68071543 | 1.557682191 | 4.04E-34 | 2.19E-31 |
| CLIC5 | 2.378239501 | 1.068380874 | -1.154467955 | 1.40E-11 | 6.02E-11 |
| CD38 | 0.910034492 | 1.849043007 | 1.022785649 | 1.35E-09 | 4.45E-09 |
| ITIH2 | 0.184806089 | 1.901786235 | 3.363270895 | 1.26E-07 | 3.28E-07 |
| TRBJ2-7 | 1.492206599 | 3.25541454 | 1.125393973 | 4.51E-15 | 3.18E-14 |
| ACAT1 | 37.37106312 | 17.35432537 | -1.106626322 | 6.43E-33 | 2.46E-30 |
| SLN | 1.012949389 | 2.314858733 | 1.192362061 | 6.12E-17 | 5.75E-16 |
| ZFPM2-AS1 | 1.063681886 | 2.236922328 | 1.072448413 | 5.37E-11 | 2.14E-10 |
| IL6 | 1.933435804 | 7.933411859 | 2.036774584 | 2.73E-17 | 2.69E-16 |
| IGKV2D-29 | 1.988395751 | 11.80316083 | 2.569496427 | 1.07E-08 | 3.17E-08 |
| ZNF683 | 0.736953979 | 1.714473697 | 1.218119336 | 1.40E-19 | 1.98E-18 |
| TRBV6-5 | 0.890876769 | 2.459634886 | 1.465146385 | 3.41E-16 | 2.83E-15 |
| TRBV2 | 0.739275867 | 1.786241859 | 1.272742713 | 1.46E-15 | 1.10E-14 |
| VTN | 2.403132934 | 5.01207246 | 1.060490816 | 3.20E-09 | 1.01E-08 |
| PPARGC1A | 5.89990637 | 2.256748692 | -1.386446288 | 2.09E-25 | 8.84E-24 |
| TNFSF14 | 0.581528304 | 2.54990659 | 2.13252308 | 1.76E-38 | 4.22E-35 |
| COL8A2 | 1.464270416 | 2.989159135 | 1.029557695 | 2.68E-13 | 1.47E-12 |
| IQSEC3 | 3.32319619 | 1.229295934 | -1.434739204 | 3.54E-07 | 8.67E-07 |
| IGKV1-16 | 8.251678725 | 31.13489984 | 1.915773081 | 3.97E-11 | 1.61E-10 |
| PAGE5 | 0.394036231 | 1.689092582 | 2.09984821 | 2.39E-07 | 5.97E-07 |
| AC061961.1 | 2.533885188 | 1.150656198 | -1.138894318 | 2.46E-18 | 2.86E-17 |
| FREM2 | 2.20690268 | 1.044921337 | -1.078628673 | 4.84E-21 | 8.91E-20 |
| AC023043.1 | 1.125018073 | 2.476699515 | 1.138470667 | 4.07E-21 | 7.64E-20 |
| FAM180A | 0.316599066 | 1.691886164 | 2.417903598 | 0.010651908 | 0.014718596 |
| MAP7D2 | 5.069389209 | 12.1533927 | 1.26147527 | 2.31E-10 | 8.45E-10 |
| SIRPG | 1.362790261 | 3.045727967 | 1.160223549 | 8.17E-21 | 1.42E-19 |
| TIMP3 | 5.61094231 | 2.552435957 | -1.136368317 | 3.19E-27 | 2.23E-25 |
| MT1X | 19.85993019 | 47.58420371 | 1.260622177 | 3.93E-10 | 1.39E-09 |
| PTH1R | 11.07601892 | 5.082150024 | -1.123928554 | 8.50E-17 | 7.73E-16 |
| UBE2QL1 | 2.643767948 | 0.890621067 | -1.56971191 | 2.13E-09 | 6.88E-09 |
| IL1R2 | 2.633822788 | 14.0614245 | 2.41651257 | 3.44E-19 | 4.60E-18 |
| IGLV2-11 | 13.43093152 | 66.09357423 | 2.298950648 | 3.40E-12 | 1.60E-11 |
| APOH | 0.953773195 | 16.91435097 | 4.148457772 | 7.98E-11 | 3.11E-10 |
| MZB1 | 1.440490589 | 8.757336421 | 2.6039319 | 9.01E-21 | 1.56E-19 |
| C8G | 0.648404338 | 1.693150088 | 1.384744216 | 5.41E-15 | 3.77E-14 |
| APOB | 0.923830363 | 4.140340144 | 2.164049426 | 1.24E-06 | 2.85E-06 |
| IGLV1-51 | 17.25037239 | 99.82127713 | 2.532719856 | 1.11E-11 | 4.86E-11 |
| SERPINE1 | 69.3127131 | 179.1132229 | 1.369679951 | 5.26E-09 | 1.61E-08 |
| CXCL1 | 4.404350416 | 12.87488561 | 1.547558454 | 6.25E-10 | 2.15E-09 |
| IGLV3-25 | 16.86311187 | 77.77492097 | 2.205434232 | 5.06E-10 | 1.76E-09 |
| NAT8B | 16.49110659 | 7.901379408 | -1.061511767 | 1.91E-12 | 9.35E-12 |
| AQP9 | 1.762471611 | 6.641076063 | 1.913817003 | 1.62E-17 | 1.66E-16 |
| NTN1 | 1.721243522 | 0.439789219 | -1.968567081 | 0.019832406 | 0.026369126 |
| PRLR | 2.087920033 | 0.737221421 | -1.501896561 | 1.57E-09 | 5.14E-09 |
| CCL21 | 7.115650989 | 20.26405618 | 1.509855326 | 1.92E-06 | 4.30E-06 |
| LIN7A | 11.03768096 | 4.507639848 | -1.291992935 | 4.67E-30 | 6.80E-28 |
| HIST2H2AC | 0.567958566 | 1.538944865 | 1.438083955 | 1.55E-10 | 5.81E-10 |
| FILNC1 | 0.873901532 | 2.204566939 | 1.334952647 | 0.000167596 | 0.000293796 |
| RNU4-1 | 3.537056356 | 19.07696086 | 2.431210244 | 0.005176355 | 0.007457171 |
| PDCD1 | 1.72835542 | 3.625145999 | 1.068639175 | 8.73E-20 | 1.26E-18 |
| CES1 | 1.77633422 | 4.881398473 | 1.458391473 | 0.001039958 | 0.001647514 |
| SLC22A12 | 23.59578528 | 8.374494308 | -1.494455204 | 1.73E-16 | 1.51E-15 |
| JCHAIN | 38.81309168 | 128.4350497 | 1.726423704 | 1.63E-11 | 6.93E-11 |
| LGALS4 | 10.65773605 | 22.17156168 | 1.056809382 | 0.000141574 | 0.000250187 |
| SLC10A2 | 8.372816999 | 2.931793833 | -1.513929438 | 8.71E-15 | 5.88E-14 |
| IGHV3-20 | 0.586633245 | 3.110465948 | 2.406599974 | 3.95E-08 | 1.09E-07 |
| TUBB4A | 4.590285884 | 2.154094934 | -1.091502175 | 0.009308353 | 0.012959833 |
| UCHL1 | 5.425205373 | 11.68343557 | 1.106714911 | 4.22E-11 | 1.70E-10 |
| TRBC2 | 11.12383238 | 25.5446851 | 1.199369241 | 2.02E-25 | 8.61E-24 |
| GABRB3 | 1.940544672 | 0.823796309 | -1.236102077 | 3.34E-15 | 2.40E-14 |
| GFPT2 | 1.338842907 | 4.75998045 | 1.829968956 | 1.35E-26 | 8.07E-25 |
| CWH43 | 1.900105426 | 0.218443701 | -3.120746056 | 7.71E-11 | 3.01E-10 |
| LRG1 | 2.91396124 | 8.106494923 | 1.476096571 | 1.37E-21 | 2.82E-20 |
| PTTG1 | 2.470794179 | 5.066549933 | 1.036028841 | 6.31E-27 | 4.17E-25 |
| TRAV21 | 0.81160849 | 1.722790319 | 1.08589126 | 4.89E-11 | 1.95E-10 |
| SNORA12 | 0.814114677 | 3.139613516 | 1.947283042 | 0.001062157 | 0.001680909 |
| C1R | 30.17803044 | 85.23221772 | 1.497900219 | 7.16E-35 | 4.64E-32 |
| PACRG | 2.250710547 | 0.755527243 | -1.574824848 | 0.000906517 | 0.001448671 |
| LUCAT1 | 0.819936424 | 2.061320673 | 1.329985001 | 9.61E-24 | 2.95E-22 |
| RNF152 | 11.95976493 | 5.502944537 | -1.11991334 | 3.73E-26 | 1.92E-24 |
| GPHN | 4.680719387 | 2.307952333 | -1.020116849 | 1.24E-29 | 1.55E-27 |
| LINC02526 | 0.66846881 | 1.453800383 | 1.120897038 | 6.76E-14 | 4.01E-13 |
| SCNN1B | 0.629805444 | 2.781992218 | 2.14314025 | 0.001960025 | 0.002991233 |
| CCL20 | 11.38234028 | 24.90563463 | 1.129674958 | 1.07E-08 | 3.17E-08 |
| FLNC | 1.732802191 | 3.522806942 | 1.023618442 | 3.87E-08 | 1.07E-07 |
| IGHV3-72 | 2.835413216 | 8.535535544 | 1.589922675 | 1.40E-06 | 3.17E-06 |
| HSD11B2 | 40.36358766 | 12.42502554 | -1.699805595 | 6.63E-17 | 6.21E-16 |
| IGHV1-2 | 7.849389717 | 45.67038228 | 2.54060647 | 6.00E-08 | 1.62E-07 |
| SH2D2A | 1.623027123 | 3.62134265 | 1.157837581 | 1.19E-29 | 1.50E-27 |
| AURKB | 0.941687045 | 2.082619327 | 1.145079571 | 1.02E-25 | 4.64E-24 |
| LRP2 | 46.44936995 | 22.78765282 | -1.027406697 | 3.23E-19 | 4.34E-18 |
| IGKV1-8 | 0.992067876 | 3.747310662 | 1.91734485 | 1.01E-14 | 6.74E-14 |
| IGHJ1 | 0.511696701 | 1.798896934 | 1.813751694 | 5.59E-06 | 1.18E-05 |
| IGHG4 | 20.12492993 | 93.20480795 | 2.211420618 | 1.21E-12 | 6.06E-12 |
| ATP8B3 | 1.06378229 | 2.42205154 | 1.187026642 | 8.85E-19 | 1.11E-17 |
| SDS | 3.523054708 | 7.377363599 | 1.066278464 | 1.84E-16 | 1.60E-15 |
| SLIT2 | 2.49955152 | 1.123213452 | -1.154037145 | 0.033711085 | 0.043134627 |
| TNNT1 | 0.453123506 | 2.011410839 | 2.150231551 | 1.41E-17 | 1.46E-16 |
| TREM1 | 0.588618041 | 1.496985394 | 1.346656479 | 2.55E-08 | 7.15E-08 |
| GPAT3 | 8.443116443 | 2.548570785 | -1.728087187 | 1.14E-29 | 1.49E-27 |
| AL590644.1 | 0.861101069 | 2.135318321 | 1.31019667 | 5.36E-11 | 2.14E-10 |
| IGKV1-17 | 6.087549718 | 40.77264146 | 2.74366787 | 1.09E-11 | 4.77E-11 |
| OXCT1 | 17.65360664 | 5.337103387 | -1.725834093 | 5.52E-21 | 1.00E-19 |
| TNFRSF18 | 0.656337572 | 1.684448751 | 1.359766609 | 1.08E-31 | 2.47E-29 |
| OACYLP | 0.271592086 | 4.975327989 | 4.195278289 | 3.11E-07 | 7.68E-07 |
| SPON1 | 26.59966012 | 9.276782289 | -1.519711423 | 4.66E-10 | 1.63E-09 |
| IGKV2-28 | 0.177405575 | 3.50996826 | 4.306334734 | 1.13E-10 | 4.34E-10 |
| CSDC2 | 5.004308407 | 2.322818937 | -1.107294002 | 5.45E-05 | 0.000101332 |
| SAA1 | 5.354145335 | 186.9148424 | 5.125581023 | 2.96E-34 | 1.67E-31 |
| REG1A | 24.28560582 | 50.32782998 | 1.051254918 | 8.73E-06 | 1.80E-05 |
| AGTR1 | 5.948590511 | 2.763474211 | -1.10606472 | 3.98E-15 | 2.82E-14 |
| SAA2-SAA4 | 0.167510894 | 7.968553228 | 5.57199098 | 1.29E-29 | 1.56E-27 |
| PPP1R14D | 3.855035609 | 11.65167738 | 1.595721569 | 1.97E-10 | 7.27E-10 |
| LINC01436 | 0.863907782 | 2.119666235 | 1.294887889 | 9.53E-17 | 8.59E-16 |
| UBE2C | 3.390565994 | 8.815910826 | 1.378583505 | 9.56E-25 | 3.58E-23 |
| CXCR3 | 2.195365725 | 4.621104284 | 1.07377635 | 2.53E-21 | 5.02E-20 |
| STEAP3 | 4.725656312 | 15.38045854 | 1.702511901 | 3.11E-22 | 7.16E-21 |
| SNORD15B | 0.938120938 | 4.475735254 | 2.254278876 | 2.72E-07 | 6.77E-07 |
| SCARNA21 | 0.948322999 | 3.969214805 | 2.06540321 | 1.99E-12 | 9.70E-12 |
| IGHV3-30 | 11.48434011 | 98.92189033 | 3.106621847 | 5.83E-10 | 2.01E-09 |
| HAUS7 | 1.099328101 | 2.385251802 | 1.117519543 | 1.02E-14 | 6.81E-14 |
| IGLC7 | 2.497173632 | 10.17351899 | 2.026450747 | 2.39E-14 | 1.52E-13 |
| IL2RA | 0.974997558 | 2.313856134 | 1.246828656 | 3.09E-10 | 1.11E-09 |
| SCARNA12 | 0.70666506 | 1.947860326 | 1.462791747 | 1.07E-05 | 2.18E-05 |
| MAP4K1 | 1.845785822 | 3.696994516 | 1.002117746 | 2.35E-26 | 1.31E-24 |
| PLA2G2D | 0.493083788 | 1.889746685 | 1.938288133 | 3.61E-18 | 4.09E-17 |
| PYCARD | 6.582994894 | 13.48527745 | 1.034569221 | 1.40E-34 | 8.59E-32 |
| TMEM252 | 15.54242572 | 7.682057289 | -1.016647056 | 3.52E-10 | 1.25E-09 |
| CXCL5 | 2.430903169 | 8.247486552 | 1.762462093 | 1.16E-13 | 6.64E-13 |
| SLAMF8 | 4.493114666 | 9.764727927 | 1.119863967 | 2.94E-19 | 4.00E-18 |
| CP | 39.56950104 | 91.45720533 | 1.208707962 | 5.12E-14 | 3.09E-13 |
| MYBL2 | 1.660664645 | 3.63628596 | 1.130704894 | 9.12E-25 | 3.43E-23 |
| IGHV4-61 | 1.495401308 | 4.584435635 | 1.616211443 | 1.01E-06 | 2.35E-06 |
| IL17RD | 2.845491776 | 1.223543583 | -1.217612519 | 3.75E-38 | 7.25E-35 |
| IGKV1D-8 | 1.002050759 | 2.752797438 | 1.457942864 | 1.40E-11 | 6.01E-11 |
| PTPN7 | 1.507822129 | 3.110922211 | 1.04487607 | 1.01E-25 | 4.60E-24 |
| IGHV3-13 | 1.732244819 | 8.328905899 | 2.265484152 | 6.78E-07 | 1.60E-06 |
| PKP1 | 1.092824237 | 2.304710897 | 1.076524404 | 0.001850721 | 0.002836908 |
| IGHV1-69D | 7.435326214 | 27.61240245 | 1.892848472 | 1.30E-09 | 4.31E-09 |
| AC015911.7 | 0.955308282 | 2.121399118 | 1.150977797 | 1.27E-20 | 2.11E-19 |
| TBC1D14 | 8.91065288 | 3.869774889 | -1.203281496 | 1.25E-18 | 1.52E-17 |
| IGLV4-69 | 4.885302505 | 69.08415692 | 3.821835091 | 4.94E-12 | 2.28E-11 |
| IGLV6-57 | 5.801786261 | 36.09827446 | 2.637360824 | 1.82E-12 | 8.93E-12 |
| SEMA3D | 1.726116159 | 0.66010229 | -1.386768046 | 2.54E-10 | 9.21E-10 |
| IGKV1-27 | 6.201782844 | 18.96282495 | 1.612418987 | 4.43E-08 | 1.21E-07 |
| ORM1 | 0.13195088 | 8.016715382 | 5.924938379 | 0.000561268 | 0.000921659 |
| SOSTDC1 | 3.631631043 | 0.793428792 | -2.194444983 | 9.32E-13 | 4.75E-12 |
| APOBEC3H | 1.054075185 | 2.168786848 | 1.040910495 | 2.59E-26 | 1.43E-24 |
| HIST1H1E | 0.456223262 | 1.664434579 | 1.867220254 | 3.41E-11 | 1.39E-10 |
| AC073346.1 | 2.288686241 | 1.063613351 | -1.105545904 | 4.58E-15 | 3.22E-14 |
| LEF1 | 1.912146167 | 3.919569091 | 1.035502247 | 2.59E-12 | 1.24E-11 |
| CTSE | 0.947979801 | 2.255914794 | 1.250784354 | 0.00040072 | 0.000671745 |
| FABP4 | 12.58246762 | 5.348996692 | -1.234074669 | 5.58E-11 | 2.22E-10 |
| SLAMF7 | 2.975813373 | 6.451900513 | 1.116440142 | 1.88E-15 | 1.40E-14 |
| AC007326.1 | 2.804829217 | 8.09672339 | 1.529425262 | 9.31E-09 | 2.77E-08 |
| TRBV4-2 | 0.742178194 | 2.141528825 | 1.528803576 | 4.05E-13 | 2.17E-12 |
| GPD1L | 8.440168129 | 4.111884299 | -1.037472068 | 2.76E-22 | 6.42E-21 |
| SNORA53 | 6.137887524 | 14.2571685 | 1.215873374 | 6.93E-05 | 0.00012728 |
| OPCML | 1.73876551 | 0.805722095 | -1.10970916 | 6.79E-09 | 2.06E-08 |
| AIM2 | 0.841286689 | 1.803023022 | 1.099748395 | 5.39E-22 | 1.20E-20 |
| TSKU | 7.579183817 | 15.2032052 | 1.004261109 | 1.20E-13 | 6.90E-13 |
| WISP2 | 0.49494995 | 2.442989095 | 2.303292874 | 6.65E-24 | 2.09E-22 |
| IL20RB | 2.937972214 | 18.02523836 | 2.617125681 | 6.53E-36 | 5.52E-33 |
| ADH1C | 0.892584463 | 1.796165709 | 1.008859856 | 0.017853394 | 0.023912682 |
| TRIM2 | 9.493717703 | 4.716343753 | -1.009304278 | 1.17E-22 | 2.95E-21 |
| CCL19 | 2.763092228 | 12.33845975 | 2.158806686 | 1.44E-15 | 1.08E-14 |
| EFNA5 | 3.515275446 | 7.803023257 | 1.150395461 | 5.61E-19 | 7.32E-18 |
| ANO4 | 2.798351788 | 1.061799341 | -1.398066187 | 2.82E-13 | 1.54E-12 |
| IGHV4-39 | 20.6729049 | 98.11567412 | 2.246742501 | 8.01E-12 | 3.59E-11 |
| IGLV1-44 | 17.03284828 | 82.50840166 | 2.276221327 | 3.60E-13 | 1.94E-12 |
| CCL18 | 7.685964475 | 16.81318834 | 1.12929512 | 9.24E-08 | 2.44E-07 |
| AC011352.3 | 2.330112195 | 5.239825735 | 1.169119409 | 4.80E-13 | 2.54E-12 |
| IGLL5 | 7.280685937 | 32.04083476 | 2.137765452 | 9.79E-16 | 7.58E-15 |
| IGLV3-16 | 0.241170786 | 6.780888381 | 4.813347231 | 8.42E-09 | 2.52E-08 |
| APOC1 | 54.50576605 | 123.9367881 | 1.185123723 | 1.53E-18 | 1.83E-17 |
| SLC16A9 | 29.46735703 | 12.04136454 | -1.291118779 | 2.14E-19 | 2.94E-18 |
| IGLV1-36 | 1.31743133 | 7.129735315 | 2.436120755 | 2.48E-11 | 1.03E-10 |
| FCGR1A | 1.944766637 | 4.168115659 | 1.099798262 | 1.45E-24 | 5.16E-23 |
| AVPR1A | 4.348849739 | 1.949651351 | -1.157417707 | 8.71E-07 | 2.03E-06 |
| CCDC91 | 4.323775815 | 9.128729325 | 1.078122336 | 1.81E-06 | 4.05E-06 |
| MYRIP | 2.076927746 | 0.65169449 | -1.672183324 | 6.29E-19 | 8.14E-18 |
| AL031429.2 | 2.749362045 | 1.316070484 | -1.062860142 | 1.11E-09 | 3.71E-09 |
| KL | 26.12210027 | 11.27626712 | -1.211981338 | 1.08E-26 | 6.75E-25 |
| IGHJ3 | 6.20455478 | 15.74089752 | 1.343118208 | 1.61E-07 | 4.10E-07 |
| IGHV2-70 | 3.78491985 | 22.81965433 | 2.591942283 | 1.15E-07 | 3.00E-07 |
| AC025265.1 | 0.782503868 | 1.734674992 | 1.148495596 | 2.51E-13 | 1.38E-12 |
| ITIH3 | 0.335600439 | 2.881353186 | 3.101930002 | 4.42E-18 | 4.96E-17 |
| IGHM | 26.72841508 | 196.5953579 | 2.878783059 | 7.09E-18 | 7.68E-17 |
| IGLC3 | 36.28127467 | 314.8535607 | 3.117383935 | 4.68E-19 | 6.19E-18 |
| MGAM | 9.70201489 | 3.970464966 | -1.288976428 | 6.43E-21 | 1.15E-19 |
| KIF20A | 1.305148552 | 2.613336919 | 1.001679109 | 5.57E-18 | 6.15E-17 |
| CNFN | 1.07300603 | 2.752823673 | 1.35925402 | 1.29E-12 | 6.47E-12 |
| MAPT | 8.438638984 | 3.602969685 | -1.227823821 | 4.97E-26 | 2.47E-24 |
| MARCO | 1.594526396 | 3.635592091 | 1.18906236 | 1.03E-06 | 2.38E-06 |
| IGHG2 | 74.80246024 | 355.237487 | 2.247626206 | 4.93E-15 | 3.46E-14 |
| HSH2D | 0.647268041 | 1.621368697 | 1.324777018 | 3.66E-26 | 1.89E-24 |
| IGKV1-39 | 0.573280034 | 1.872162602 | 1.707393803 | 1.39E-09 | 4.59E-09 |
| SLC17A9 | 1.180985801 | 2.945847449 | 1.318691102 | 1.87E-20 | 3.03E-19 |
| TOX3 | 2.21754074 | 0.615512607 | -1.849100298 | 2.37E-18 | 2.77E-17 |
| RFLNA | 0.591903046 | 2.446414241 | 2.047235923 | 2.03E-12 | 9.89E-12 |
| PI3 | 0.774942898 | 7.512768929 | 3.277182817 | 3.02E-23 | 8.46E-22 |
| HSD11B1 | 0.929823789 | 2.822684837 | 1.602038814 | 1.13E-12 | 5.68E-12 |
| IGHV3-48 | 2.687524323 | 16.26497705 | 2.59741907 | 1.32E-09 | 4.35E-09 |
| IGKV3-15 | 9.262844604 | 70.85593202 | 2.935361424 | 6.30E-13 | 3.28E-12 |
| TGFBI | 99.39765057 | 342.4418816 | 1.784575501 | 2.70E-21 | 5.32E-20 |
| CCL5 | 32.79018525 | 71.911905 | 1.132966576 | 2.89E-20 | 4.53E-19 |
| APOA1 | 0.218440264 | 3.342048068 | 3.935421773 | 7.01E-06 | 1.46E-05 |
| SLC34A2 | 8.10262669 | 22.48046632 | 1.472210384 | 1.69E-11 | 7.16E-11 |
| LINC00626 | 0.94152101 | 3.209613401 | 1.76933434 | 9.60E-05 | 0.000173617 |
| SLC38A5 | 1.346462471 | 6.27377303 | 2.220159318 | 9.72E-29 | 1.04E-26 |
| TRAC | 13.80658726 | 27.88882467 | 1.01433038 | 1.76E-20 | 2.87E-19 |
| HMGCS2 | 26.43550708 | 8.179178905 | -1.692449075 | 6.35E-14 | 3.78E-13 |
| ITPKA | 0.479167102 | 2.449238641 | 2.353732582 | 2.30E-19 | 3.16E-18 |
| AL139349.1 | 1.075410786 | 2.221764448 | 1.046818023 | 6.89E-10 | 2.36E-09 |
| C2 | 5.799802336 | 12.3248055 | 1.087489242 | 1.19E-17 | 1.25E-16 |
| AC015722.2 | 3.315277542 | 8.51257817 | 1.360466489 | 1.59E-09 | 5.21E-09 |
| APCDD1L | 0.698477371 | 2.017167235 | 1.530045415 | 6.32E-11 | 2.49E-10 |
| PAEP | 0.312796877 | 13.38030785 | 5.418741389 | 2.78E-20 | 4.36E-19 |
| IGLV1-41 | 0.311005731 | 2.318603378 | 2.898242984 | 1.32E-09 | 4.37E-09 |
| CYP4A22 | 3.576422893 | 1.383967614 | -1.369707155 | 3.32E-11 | 1.36E-10 |
| SNCG | 12.94862469 | 26.25316901 | 1.019692707 | 3.71E-10 | 1.31E-09 |
| IGKV1-12 | 0.998183151 | 2.602372271 | 1.382450898 | 8.77E-12 | 3.90E-11 |
| IGHV4-34 | 8.283113005 | 46.79653793 | 2.498156825 | 4.20E-12 | 1.96E-11 |
| ACHE | 0.879803628 | 2.204580282 | 1.32525056 | 5.57E-18 | 6.15E-17 |
| IGHV3-15 | 14.3237966 | 61.28477864 | 2.097114857 | 2.10E-10 | 7.73E-10 |
| SIM1 | 1.683508927 | 0.596407805 | -1.497100327 | 1.92E-09 | 6.21E-09 |
| MMP19 | 1.387410477 | 2.999301447 | 1.112231844 | 6.94E-19 | 8.90E-18 |
| AP001453.2 | 0.741071055 | 1.966648771 | 1.408055544 | 8.06E-20 | 1.17E-18 |
| WFDC5 | 0.281370797 | 1.855457156 | 2.72123018 | 2.71E-17 | 2.68E-16 |
| ITGB2-AS1 | 0.943845111 | 2.383343708 | 1.33636499 | 2.94E-20 | 4.59E-19 |
| PLG | 4.930914121 | 1.810234427 | -1.445678586 | 1.04E-06 | 2.41E-06 |
| GACAT2 | 1.815653072 | 4.444311271 | 1.291471299 | 5.53E-06 | 1.17E-05 |
| NR3C2 | 3.675967052 | 1.609653171 | -1.191373969 | 4.99E-31 | 9.92E-29 |
| AC116345.3 | 3.53022815 | 1.556307821 | -1.181633987 | 4.50E-16 | 3.64E-15 |
| FDCSP | 0.221433459 | 18.16589754 | 6.358215603 | 3.13E-13 | 1.70E-12 |
| IGHV6-1 | 0.668204615 | 4.396509864 | 2.717996854 | 5.03E-09 | 1.55E-08 |
| IGKV2D-40 | 1.279390264 | 4.050560288 | 1.66266507 | 7.38E-09 | 2.22E-08 |
| IGHV3-43 | 1.105443991 | 8.297328407 | 2.908020958 | 1.70E-10 | 6.35E-10 |
| SRPX2 | 2.193502706 | 4.798896688 | 1.129466269 | 2.01E-17 | 2.03E-16 |
| S100G | 0.118952508 | 3.056136004 | 4.683251158 | 8.45E-08 | 2.24E-07 |
| AVPR1B | 2.507076573 | 1.16093801 | -1.110715121 | 1.45E-11 | 6.22E-11 |
| IGHV1-46 | 5.656461388 | 26.2413084 | 2.213867947 | 8.14E-09 | 2.44E-08 |
| IGKC | 116.3174032 | 764.0387956 | 2.71557893 | 7.50E-19 | 9.57E-18 |
| IGKV3-7 | 0.783084513 | 2.60843843 | 1.735946459 | 3.22E-08 | 8.97E-08 |
| STUM | 2.198406756 | 0.840609828 | -1.386950114 | 9.23E-17 | 8.34E-16 |
| IGHV4-28 | 1.014829609 | 9.177510815 | 3.176865392 | 4.00E-11 | 1.62E-10 |
| CPNE7 | 0.528639108 | 1.946278896 | 1.880363397 | 7.61E-27 | 4.97E-25 |
| IGLV3-1 | 22.6815611 | 55.31006247 | 1.286022031 | 1.08E-11 | 4.74E-11 |
| SNORD99 | 3.129636711 | 8.988015192 | 1.522007365 | 1.02E-20 | 1.74E-19 |
| TFPI2 | 16.13510857 | 32.8118292 | 1.024012738 | 1.33E-06 | 3.02E-06 |
| HIST1H4E | 1.07193068 | 2.530848838 | 1.239409729 | 9.57E-10 | 3.22E-09 |
| AL355075.4 | 0.487721175 | 2.077335892 | 2.090605993 | 1.04E-05 | 2.12E-05 |
| C16orf74 | 1.826372168 | 6.277205429 | 1.781141644 | 1.18E-28 | 1.23E-26 |
| DNER | 0.912822275 | 2.237079114 | 1.293210377 | 8.39E-12 | 3.75E-11 |
| ADAMDEC1 | 1.584915629 | 3.776755909 | 1.252741503 | 9.71E-14 | 5.63E-13 |
| IGHD | 3.989633085 | 12.10207507 | 1.600926461 | 1.01E-05 | 2.05E-05 |
| FAM189A2 | 1.739424519 | 0.813194313 | -1.096938046 | 5.29E-05 | 9.86E-05 |
| SNORD17 | 6.682569084 | 45.88810368 | 2.779645437 | 0.000349903 | 0.000590123 |
| BSPRY | 3.489412841 | 1.726302079 | -1.015299358 | 6.79E-13 | 3.52E-12 |
| SCARNA5 | 2.616090345 | 34.5863321 | 3.724717754 | 3.63E-05 | 6.91E-05 |
| IGHV1-12 | 0.320457265 | 1.767257043 | 2.463308008 | 3.86E-09 | 1.20E-08 |
| IGHJ3P | 0.950232283 | 4.810925202 | 2.339962243 | 1.49E-13 | 8.47E-13 |
| MUC20 | 14.56640196 | 4.376717158 | -1.734723503 | 0.000425957 | 0.000710681 |
| AC156455.1 | 1.204324786 | 2.725692665 | 1.178398386 | 5.35E-25 | 2.09E-23 |
| RNASE2 | 1.21888404 | 2.847112261 | 1.223938499 | 5.35E-24 | 1.70E-22 |
| LINC01127 | 1.977962185 | 8.196490794 | 2.050991529 | 3.12E-15 | 2.25E-14 |
| SNORD100 | 1.251542624 | 2.798823385 | 1.161113025 | 2.19E-14 | 1.40E-13 |
| TBC1D10C | 1.552113583 | 3.187594715 | 1.038234074 | 6.45E-25 | 2.46E-23 |
| FOSB | 44.27368847 | 21.86690947 | -1.017700237 | 0.000482869 | 0.000799619 |
| SLC16A12 | 29.34126275 | 13.67050846 | -1.101864058 | 9.78E-21 | 1.67E-19 |
| IGHV3-7 | 0.875017094 | 4.113787247 | 2.233084077 | 3.20E-10 | 1.14E-09 |
| IGHV3-23 | 23.28723682 | 129.9547886 | 2.480398425 | 8.11E-10 | 2.75E-09 |
| SAA2 | 0.472440883 | 14.8783251 | 4.976934502 | 3.47E-31 | 7.34E-29 |
| SLC5A1 | 16.84461267 | 5.68260469 | -1.567662993 | 3.85E-15 | 2.74E-14 |
| LBP | 3.134903784 | 53.75949845 | 4.100026609 | 2.91E-23 | 8.22E-22 |
| C4orf48 | 1.488245574 | 3.615570389 | 1.28061066 | 3.09E-16 | 2.58E-15 |
| ALDH6A1 | 13.46569581 | 5.626311845 | -1.259027357 | 6.09E-42 | 8.23E-38 |
| TRHDE | 2.46430724 | 1.213215919 | -1.022345804 | 7.02E-23 | 1.86E-21 |
| AC010970.1 | 0.59888193 | 1.550188597 | 1.372100237 | 0.000561423 | 0.000921803 |
| MELTF | 0.657992479 | 1.73155032 | 1.395921315 | 6.57E-24 | 2.07E-22 |
| SCARNA13 | 2.851227644 | 6.09510581 | 1.096068036 | 0.022880519 | 0.030084757 |
| JAK3 | 3.135383169 | 6.824607816 | 1.12210438 | 1.87E-38 | 4.22E-35 |
| AL591845.1 | 1.69012817 | 3.436466706 | 1.023793325 | 7.93E-16 | 6.22E-15 |
| AC026403.1 | 3.935768097 | 8.338471396 | 1.083137717 | 4.27E-13 | 2.28E-12 |
| IGHV5-51 | 19.6734571 | 95.49985179 | 2.279247998 | 1.33E-10 | 5.06E-10 |
| PLTP | 26.00928724 | 74.42035127 | 1.516670336 | 3.95E-23 | 1.09E-21 |
| SCGN | 42.19760917 | 18.73677348 | -1.171288722 | 1.01E-15 | 7.81E-15 |
| LIX1 | 2.320734591 | 0.817734467 | -1.504877184 | 0.000138958 | 0.000245992 |
| C19orf33 | 30.82698076 | 65.96957264 | 1.097607162 | 7.68E-15 | 5.22E-14 |
| GCNT4 | 1.484822656 | 0.738498397 | -1.007623934 | 9.54E-11 | 3.68E-10 |
| C1QL1 | 13.27618222 | 41.12157193 | 1.631055078 | 1.09E-18 | 1.34E-17 |
| AP005233.2 | 2.052457731 | 5.954467085 | 1.536619884 | 2.71E-18 | 3.14E-17 |
| DSG2 | 35.61244878 | 17.80324623 | -1.000241316 | 1.23E-26 | 7.48E-25 |
| TNNT3 | 0.462588646 | 1.973879712 | 2.093232312 | 0.009401611 | 0.013081398 |
| EPS8L3 | 0.767114442 | 2.277744374 | 1.570092118 | 1.46E-06 | 3.32E-06 |
| IGKV1-6 | 6.748943345 | 20.80977779 | 1.624528012 | 4.41E-10 | 1.55E-09 |
| SYT9 | 2.58162851 | 1.247474503 | -1.049271086 | 5.22E-11 | 2.08E-10 |
| SERPINF1 | 10.51772819 | 27.87108377 | 1.405945984 | 2.52E-32 | 7.25E-30 |
| AC116614.1 | 0.200291501 | 2.506880703 | 3.645720237 | 5.05E-26 | 2.49E-24 |
| MOCOS | 0.626745902 | 2.017059593 | 1.686301144 | 2.56E-27 | 1.85E-25 |
| IGKV3-20 | 42.71043383 | 284.751681 | 2.737043902 | 2.09E-13 | 1.16E-12 |
| LINC01857 | 0.766814333 | 1.600728042 | 1.06177901 | 3.71E-13 | 2.00E-12 |
| AL138826.1 | 6.43790561 | 3.055754634 | -1.07506272 | 1.29E-08 | 3.77E-08 |
| SNORA73B | 4.902464348 | 66.31785249 | 3.757818246 | 2.35E-14 | 1.49E-13 |
| CD177 | 0.236831824 | 2.833880342 | 3.580843987 | 1.02E-08 | 3.01E-08 |
| IGLV5-45 | 1.848274659 | 9.836059589 | 2.411901314 | 2.38E-07 | 5.96E-07 |
| PLA2G2A | 0.201587438 | 1.966200474 | 3.285932786 | 8.00E-15 | 5.42E-14 |
| SNORA20 | 0.495307704 | 1.996892046 | 2.011359376 | 0.001886506 | 0.002885871 |
| FCHO1 | 0.71584685 | 1.555616557 | 1.119763624 | 2.05E-32 | 6.30E-30 |
| MFSD2A | 0.613359904 | 2.574891677 | 2.069705976 | 4.13E-27 | 2.85E-25 |
| IGLV2-23 | 22.73770358 | 132.1441114 | 2.538953676 | 4.34E-11 | 1.75E-10 |
| IGHV3-64 | 0.687062757 | 2.981940709 | 2.117737785 | 1.93E-08 | 5.52E-08 |
| SNORA7B | 0.671380904 | 1.738996504 | 1.373051624 | 0.00014976 | 0.000264066 |
